# Numerical Investigation of a Skin-Interfaced Thermal Sensor for Joint Estimation of Tissue Thermal Conductivity and Blood Velocity

**DOI:** 10.3390/mi17091093

**Published:** 2026-09-17

**Authors:** Lifei Qi, Tianyu Yang

**Affiliations:** School for Engineering of Matter, Transport and Energy, Arizona State University, Tempe, AZ 85281, USA; lifeiqi@asu.edu

**Keywords:** biosensor, heat transfer, skin, flow, machine learning, simulation, reduced-order model

## Abstract

Skin-interfaced thermal sensors offer a promising, portable, and cost-effective alternative for continuous and noninvasive measurements of skin condition and blood flow. Skin condition, especially skin hydration, is reflected by the tissue thermal conductivity. Blood flow is characterized by the average flow velocity through blood vessels in skin. However, the measurement accuracy of tissue thermal conductivity and blood velocity is hindered by the coupled heat conduction and convection in the tissue containing blood vessels. To overcome this bottleneck for precise measurements of tissue thermal conductivity and blood velocity simultaneously, we design a skin-interfaced thermal sensor consisting of a resistive heater and three thermistors. The resistive heater with a diameter of 4 mm consumes a low power of 0.05 W. The three miniature thermistors measure the steady-state temperatures on skin at the middle location of the heater center, upstream flow location, and downstream flow location. Using finite element analysis (FEA) of heat transfer in vascular skin, we optimize the three-thermistor layout, placing the upstream and downstream thermistors 6.0 mm and 2.7 mm from the heater center, respectively. FEA results reveal that the middle-thermistor temperature is predominantly sensitive to tissue thermal conductivity with relatively low flow interference, whereas the temperature difference between upstream and downstream thermistors maintains high sensitivity to blood velocity, and is less affected by tissue thermal conductivity. With the FEA results, we implement a polynomial machine learning model and a physics-informed thermal-resistance reduced-order model to analyze the thermal sensor temperature measurements and jointly predict both quantities. The relative prediction errors are typically below 4% for thermal conductivity and 10% for blood velocity using the machine learning model, and below 1% and 8% using the reduced-order model. This work provides a framework for the development of skin-interfaced thermal sensors capable of intelligent and noninvasive skin and vascular assessment.

## 1. Introduction

Recent advances in flexible sensing continue to expand design options for skin-interfaced sensors. Representative developments include tactile sensors [1], large-deformation fabric sensors [2], and adaptive polymer materials [3]. Different forms of wearable sensors enable various thermal sensing platforms by leveraging microfabrication techniques and microscale thermal-fluid transport principles for noninvasive physiological assessment [4,5]. At the component level, such devices can be fabricated using microscale resistive heater traces patterned and etched on flexible printed circuit boards (F-PCBs) with total thicknesses below 100 μm. Commercially available 0201 NTC thermistors have dimensions of approximately 600 μm × 300 μm × 100 μm, providing microscale temperature-sensing elements compatible with these flexible platforms. Together, these miniaturized components can be integrated into soft, flexible microsystem architectures to enable wearable micro-thermal sensors that provide multiparameter physiological information with high precision and low power consumption [6,7].

Continuous and noninvasive monitoring of superficial skin and vascular conditions by wearable sensors is critical for the early detection of local physiological and pathological changes. Blood flow velocity is a key indicator of local perfusion and vascular function, and abrupt changes in blood flow may signal vascular complications such as stenosis, thrombosis, or impaired tissue viability [8,9]. Tissue thermophysical properties, including thermal conductivity and thermal diffusivity, are also important because they depend on tissue composition and water content and may change with skin dehydration, edema, inflammation, and other alterations in the local tissue environment [10,11,12,13,14]. Therefore, accurate characterization of both blood flow and tissue thermophysical properties can provide valuable information about vascular and skin conditions. Conventional blood flow measurement techniques, such as Doppler ultrasound and photoacoustic imaging, can provide detailed vascular information but commonly rely on specialized or relatively bulky instrumentation, coupling media, and controlled probe positioning [15]. Measurements of tissue thermophysical properties also generally require dedicated equipment and controlled measurement conditions. These limitations make conventional techniques difficult to use for convenient, long-term, and wearable monitoring.

To address these limitations, wearable micromachined thermal sensors offer a promising alternative for continuously assessing both vascular and tissue properties [16,17]. These sensors apply a controlled, low-level heat input to the skin surface. The resulting skin temperature response is then monitored as heat propagates through the tissue. In a static tissue environment, the applied heat dissipates solely via thermal conduction through the skin. When blood flows through an adjacent vessel, it acts as a convective medium that transports thermal energy along the direction of the flow. Existing wearable thermal devices generally employ either steady-state spatial temperature measurements or transient thermal analysis to characterize this conjugate heat transfer process [8,9,18]. Steady-state spatial methods measure temperature differences around the heater, whereas transient methods analyze the time-dependent temperature decay. Tissue thermal conductivity *k*_s_ governs the ability of heat to diffuse through the tissue and determines its conductive thermal resistance, whereas blood velocity, *V*_f_, controls the capacity of flowing blood to dilute thermal energy. During active thermal measurements, conduction through the tissue and convective transport by the blood flow occur simultaneously, so the skin surface temperature response is jointly influenced by *k*_s_ and *V*_f_. Because conventional sensors cannot separate these two contributions, predictions of either parameter can be highly inaccurate.

Prior works have addressed this thermal coupling using various sensing strategies. Sequentially combined measurements of local tissue properties, vessel depth, and upstream–downstream temperature asymmetry have been used to estimate blood velocity and direction in superficial vessels [9]. To determine background tissue properties before estimating blood flow, a separate tissue location was used, causing risks of location- and time-dependent property change [10]. To infer blood velocity from temperature measurements, an analytical model incorporating heat conduction and convection was applied [18]. However, its reliance on thermal conductivity obtained via blood flow occlusion adds operational complexity and introduces measurement errors. Transient hot-film and calorimetric methods have also been developed to improve sensitivity to high biofluid flow rates by analyzing peak temperature responses [19]. In these vascular thermal flow sensing strategies, tissue thermal conductivity *k*_s_ is typically treated as a known material property, a separately calibrated parameter, or a preliminary measurement used to support subsequent flow estimation, rather than as a simultaneous prediction target together with blood velocity *V*_f_. Moreover, it remains unclear whether the measurement of temperature can jointly predict both parameters with acceptable accuracy. The decoupling of *k*_s_ and *V*_f_ is critical to minimize the prediction error for accurate assessment of skin and vascular conditions. Furthermore, simultaneous measurement at one location saves the time and effort of patients and healthcare workers.

This paper describes the design and numerical investigation of a skin-interfaced thermal sensor consisting of a central heater and three in-line thermistors for simultaneous measurement of tissue thermal conductivity *k*_s_ and blood velocity *V*_f_. The thermal sensor is attached to the skin surface, with the three thermistors aligned along a blood vessel located 1.7 mm beneath the surface. The central heater on top of the blood vessel has a diameter of 4 mm and generates heat at a low power of 0.05 W. Three miniature thermistors measure the steady-state skin temperature at the heater center and at upstream and downstream locations along the flow direction. A three-dimensional finite element analysis (FEA) model of conjugate heat transfer in vascularized skin is developed to quantify parameter sensitivities and optimize the thermal sensor design. The model is further used to simulate steady-state temperature responses to various *k*_s_ (0.20~0.40 W/(m·K)) and *V*_f_ (0.01~0.10 m/s). To measure *k*_s_ and *V*_f_ directly from the thermal sensor readings, we establish two distinct prediction frameworks: a data-driven machine learning (ML) model and a physics-based reduced-order model (ROM) constructed from a thermal-resistance network. Both models are trained by FEA results and simultaneously predict *k*_s_ and *V*_f_ based on inputs of thermistors’ temperature measurements. The relative prediction errors are typically below 4% for *k*_s_ and 10% for *V*_f_ using the ML model and below 1% and 8% using the ROM.

## 2. Methods

### 2.1. Finite Element Analysis Model

A three-dimensional steady-state finite element model was developed in COMSOL Multiphysics 6.2 to couple laminar blood flow with heat transfer in the blood and surrounding tissue. The vessel was modeled as a circular tube with a radius *r*_v_ = 1.3 mm and a centerline depth *h*_v_ = 1.7 mm below the tissue surface. The heater and thermistor radii were 2.0 and 0.5 mm, respectively. The thermophysical properties used in the simulations are listed in Supplementary Table S1. Tissue thermal conductivity *k*_s_ was varied from 0.20 to 0.40 W/(m·K) in increments of 0.02 W/(m·K), while the mean inlet velocity *V*_f_ was varied from 0.01 to 0.10 m/s in increments of 0.01 m/s, resulting in 110 parameter combinations. At the vessel inlet, a fully developed laminar flow condition was applied and the blood temperature was set to 37 °C. At the outlet, the static gauge pressure was set to 0 Pa, with backflow suppression enabled. A no-slip condition was applied at the vessel wall. Temperature and heat flux were continuous across the tissue–blood interface, and the body-temperature boundaries were maintained at 37 °C. The heater supplied a constant power of *Q* = 0.05 W. Natural convection was applied to the exposed tissue surface using heat transfer coefficient *h*_e_ = 5 W/(m^2^·K) and an ambient temperature *T*_e_ = 25 °C. These values were selected as representative of low-air-movement indoor conditions, consistent with previous thermal models of skin and skin-interfaced electronics [20,21]. The convective heat loss was calculated locally using Newton’s law of cooling based on the temperature difference between the exposed surface and the ambient environment. The temperatures of the three thermistors were obtained by area-averaging the calculated surface temperatures over their respective sensing regions.

### 2.2. Machine Learning

A supervised machine learning framework was developed to predict *k*_s_ and *V*_f_ from the three thermistor temperatures *T*_u_, *T*_m_, and *T*_d_. The dataset comprised the 110 FEA cases generated from the full-factorial parameter sweep, with the three area-averaged temperatures used as inputs and *k*_s_ and *V*_f_ used as outputs. Linear regression, second- and third-order polynomial ridge regression (P3), radial-basis-function support-vector regression, Extra Trees, distance-weighted k-nearest neighbors, and Random Forest were compared using shuffled five-fold cross-validation. Model performance was evaluated from out-of-fold predictions using the mean absolute error, root-mean-square error, coefficient of determination, and root-mean-square error normalized by the full sampled range of each output. The third-order polynomial ridge model produced the lowest mean normalized error of 0.269% and was selected for subsequent predictions (Supplementary Table S2). The selected pipeline standardized the temperature inputs before third-order polynomial expansion and applied ridge regression with a regularization coefficient *α =* 10^−6^. The final model was refitted using all 110 FEA cases. Model generalization was additionally evaluated using 30 independent off-grid *k*_s_ and *V*_f_ combinations generated by maximin Latin-hypercube sampling within the original parameter bounds. New FEA simulations were performed for these cases, and the selected model was kept fixed throughout the external validation.

### 2.3. Thermal-Resistance Network Analysis

A reduced-order thermal-resistance model (ROM) was constructed to describe heat transfer from the heater to the surrounding tissue, blood flow, and environment. The thermistor locations were represented by the surface nodes u, m, and d, while the blood and deep tissue were treated as thermal reservoirs at *T*_b_. The network includes lateral surface conduction, conduction from the surface to the vessel followed by blood convection, bypass conduction into deeper tissue, and natural-convection heat loss to the environment. A spreading resistance was introduced at node m to relate the measured heater-center temperature *T*_m_ to the effective subsurface temperature.

The model parameters were determined using physical relations and FEA results. The conductive and spreading resistances were formulated as functions of *k*_s_. The effective lateral and bypass conduction areas were determined from the FEA results (Supplementary Figures S1 and S2). Because *T*_u_ showed limited variation over the investigated parameter space (Supplementary Figure S3), node u was represented as a lumped temperature node. For the blood convection branches, mean heat transfer coefficients over the axial intervals represented by nodes m and d were obtained using the Hausen correlation for hydrodynamically fully developed but thermally developing laminar flow [22]. These coefficients were subsequently converted to convective thermal resistances. The ROM parameters were calibrated by minimizing the differences between the ROM-calculated and FEA-derived *T*_m_ and *T*_d_. The calibrated ROM was then used to predict *k*_s_ and *V*_f_ from the three sensor temperatures. Complete resistance definitions, governing equations, calibration procedures, and prediction algorithms are provided in Supplementary Section S1.

## 3. Results

**Figure 1:**
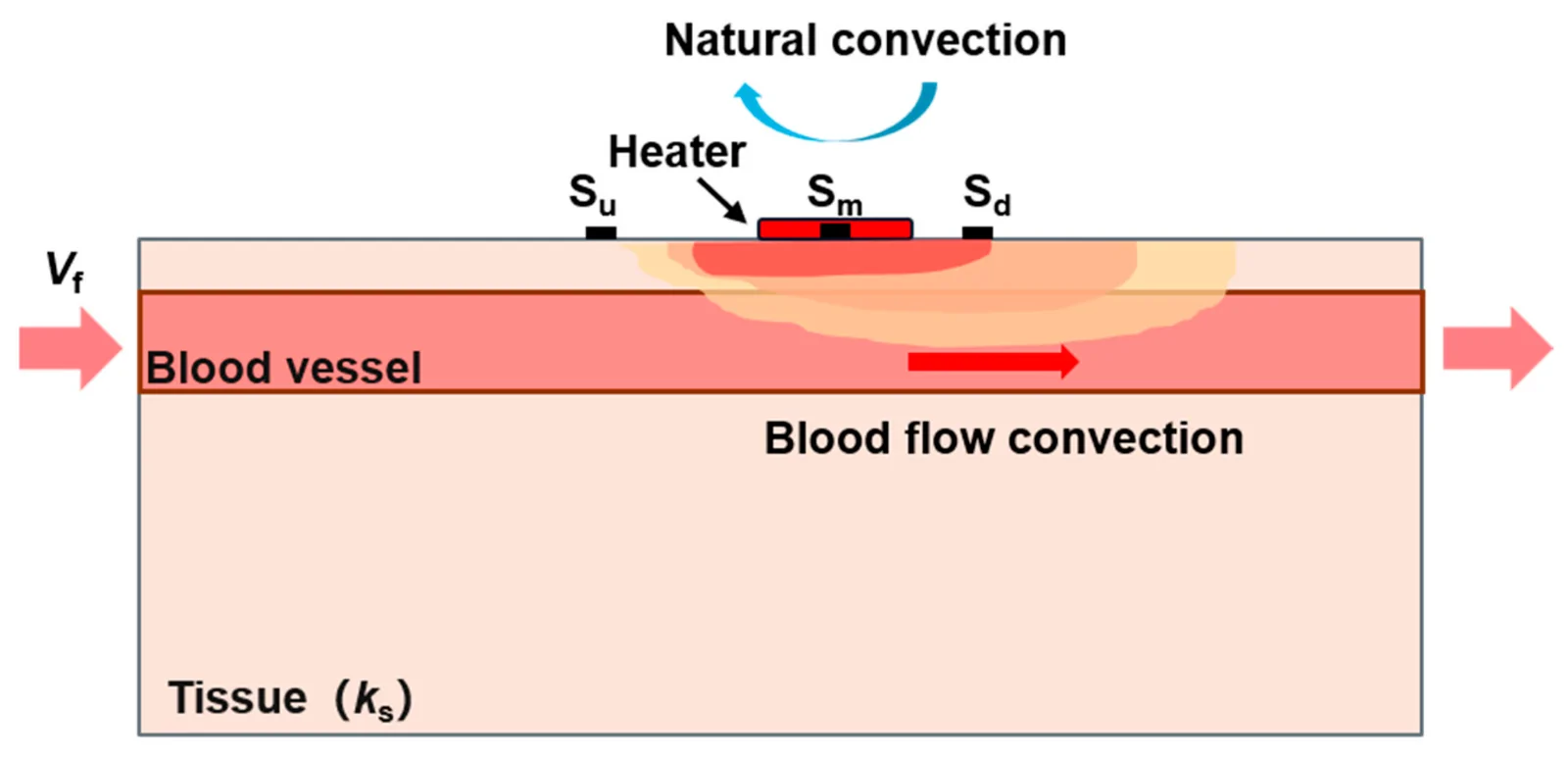
Schematic illustration of the thermal sensor consisting of a heater and three temperature sensors (S_u_, S_m_ and S_d_) on a skin tissue with an underlying vein. The thermal sensor is designed to measure the blood flow velocity *V*_f_ and the tissue thermal conductivity *k*_s_. The heater generates a power of *Q* = 0.05 W. The color gradient beneath the heater schematically illustrates heat diffusion from the heater into the skin tissue.

Figure 1 illustrates the configuration and operating principle of the proposed thermal sensor. The thermal sensor consists of a resistive heater and three thermistors, S_u_, S_m_, and S_d_, positioned on the skin surface with a blood vessel 1.7 mm deep in the skin tissue, consistent with the relative deep wrist vein reported by Webb et al. [9]. S_u_ measures the upstream skin temperature affected by environment and heater, S_d_ measures the downstream temperature affected by the blood flow dilution, and S_m_ in heater center measures the heater temperature. A constant heating power of 0.05 W is applied to generate a localized thermal perturbation in the tissue with a heat flux of 3.979 mW/mm^2^. The resulting temperature field is jointly governed by heat conduction through the surrounding tissue and convective heat dilution by the blood flow. Tissue thermal conductivity *k*_s_ controls the lateral and vertical spreading of heat away from the heater and therefore influences the magnitude and spatial extent of the surface temperature rise. By contrast, blood flow transports heat along the vessel, producing a directional thermal response and a temperature difference between the upstream and downstream sensing locations. Convective heat transport increases the downstream temperature relative to the upstream temperature. The three-thermistor arrangement is designed to capture these different thermal transport characteristics. Therefore, the combined temperature measurements *T*_u_, *T*_m_, and *T*_d_ contain distinguishable information associated with *k*_s_ and *V*_f_, enabling the two coupled physiological parameters to be simultaneously determined using a single heating event.

**Figure 2:**
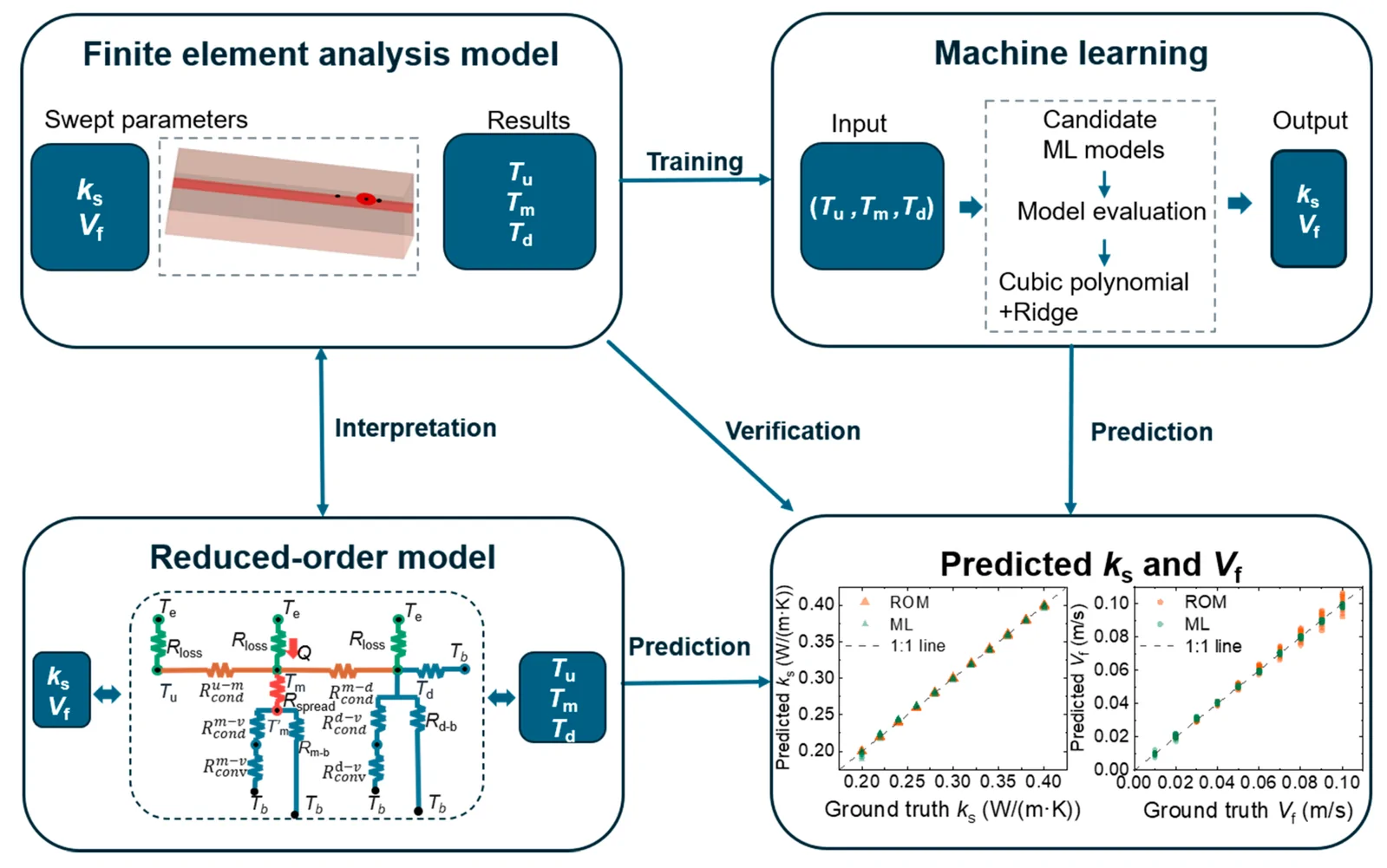
Integrated workflow for the thermal sensor design, optimization, and performance characterization.

Figure 2 shows the integrated framework to design, analyze, and evaluate the proposed thermal sensor. First, a three-dimensional FEA model was established to simulate heat transfer within the tissue and blood vessel, with tissue thermal conductivity *k*_s_ ranging from 0.2 to 0.4 W/(m·K), and blood velocity *V*_f_ ranging from 0.01 to 0.10 m/s. The *k*_s_ range was centered on a representative tissue thermal conductivity of 0.3 W/(m·K), and the upper limit of *V*_f_ was set at 0.10 m/s, consistent with reported peripheral venous velocities [9,23,24]. For each parameter combination, the model calculated the temperatures *T*_u_, *T*_m_, and *T*_d_ at the three thermistor locations, thereby generating a temperature-parameter dataset for evaluating the thermal sensor response. Two complementary approaches were then developed to predict *k*_s_ and *V*_f_ from the three thermistor temperatures. In the machine learning approach, the FEA-generated dataset was used to train and evaluate candidate regression models. A cubic polynomial regression model with ridge regularization was selected to establish the prediction algorithm from *T*_u_, *T*_m_, and *T*_d_ to *k*_s_ and *V*_f_. This data-driven approach captures the nonlinear relationships between the three thermistor responses and the two target parameters. In parallel, a physics-based reduced-order model was constructed to elucidate heat transfer mechanisms by a thermal-resistance network including heat conduction through the tissue, convective heat transfer to the blood flow, lateral heat spreading between sensing regions, and heat loss to the environment. The thermal resistances and velocity-dependent parameters were calibrated and validated using the FEA results. The ROM therefore provides both a computationally efficient prediction method and a physical interpretation of how *k*_s_ and *V*_f_ influence the measured temperatures. The ML and ROM approaches use the same three thermistor temperatures but determine *k*_s_ and *V*_f_ through fundamentally different representations of the heat transfer process. Their predictions were compared with the corresponding FEA ground-truth values. Agreement among the ML, ROM, and FEA results evaluates not only the predictive models but also whether the proposed thermal sensor provides sufficient and distinguishable thermal information for simultaneously determining tissue thermal conductivity and blood velocity.

**Figure 3:**
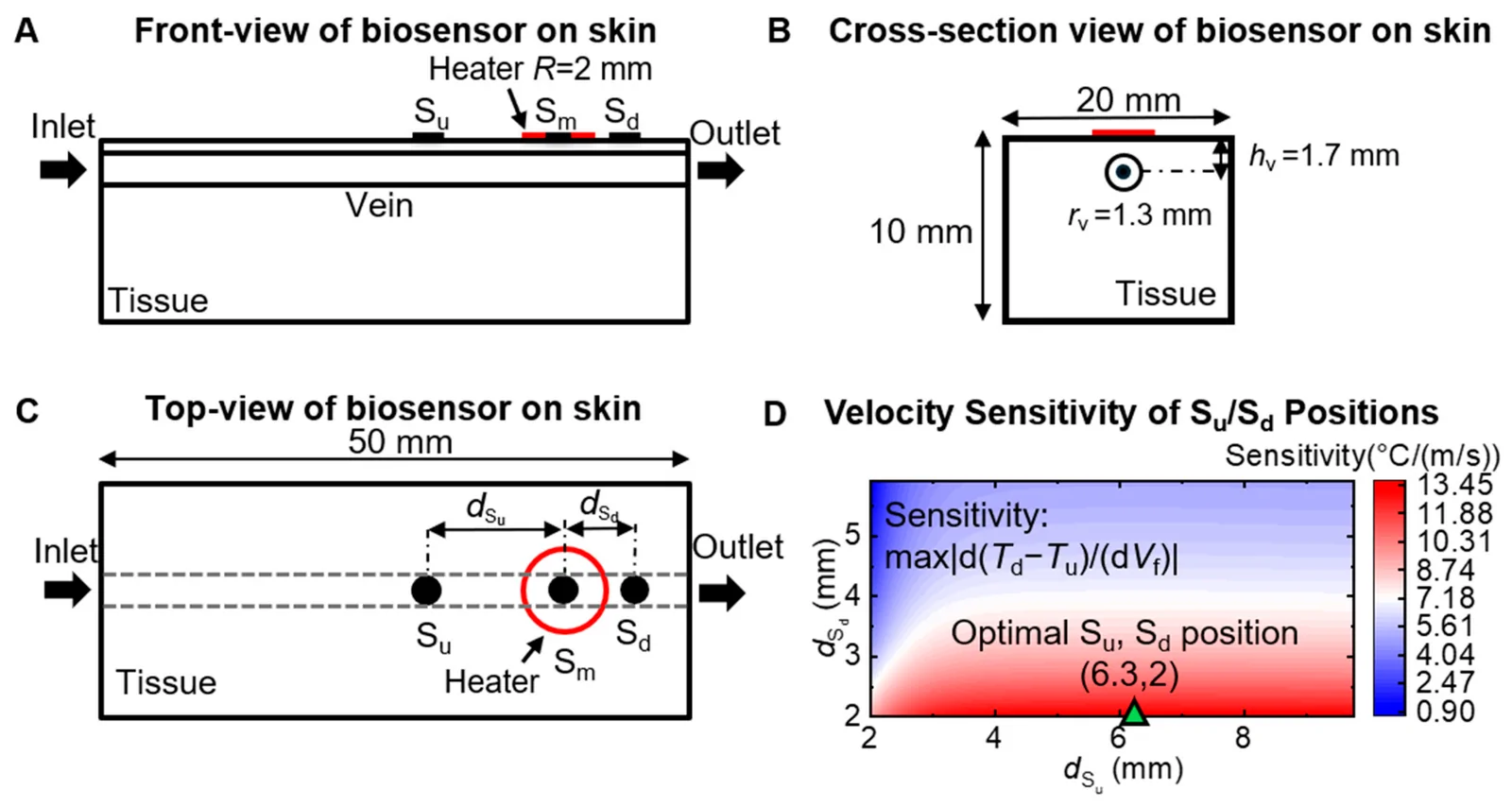
Geometric configuration of the FEA model and spatial optimization of the thermal sensor layout. (**A**) Front-view of the thermal sensor configuration. (**B**) Cross-sectional view showing the geometric dimensions. (**C**) Top-view of the thermal sensor arrangement with thermistor radius *r*_s_ = 0.5 mm. (**D**) Point-based sensor-pair sensitivity map of max |d(*T*_d_ − T_u_)/d*V*_f_| with respect to *V*_f_. The green triangle indicates optimal thermistor location. The implemented realistic sensor-center distances are *d*_S*u*_ = 6.0 mm, *d*_S*d*_ = 2.7 mm, after accounting for the thermistor size.

To evaluate the thermal response and optimize the configuration of the thermal sensor, a three-dimensional steady-state FEA model was developed in COMSOL Multiphysics 6.2. The model couples heat conduction in the tissue with convective heat transport by blood flow, thereby representing the dominant heat transfer processes of the physical system. The parameters used in the FEA model are summarized in Supplementary Table S1. Figure 3 shows the geometric configuration of the FEA model and the layout optimization of the thermal sensor. The thermal sensor was positioned above a subsurface blood vessel and consisted of a circular heater with a radius of 2 mm and three thermistors with a radius of 0.5 mm. The modeled tissue domain had a length of 50 mm, a width of 20 mm, and a height of 10 mm. The vessel had a radius of 1.3 mm and its center was located 1.7 mm beneath the tissue surface, resulting in a minimum tissue thickness of 0.4 mm between the vessel wall and the skin surface [9]. Heat from the thermal sensor must conduct through the tissue before blood convection, leading to strong conjugate heat transfer between the surface heater and the blood flow. S_m_ was located at the heater center, and S_u_ and S_d_ were positioned upstream and downstream of the heater along the vessel axis. The locations of S_u_ and S_d_ were optimized by maximizing the absolute sensitivity of the differential temperature to blood flow velocity, that is, |d(*T*_d_ − T_u_)/d*V*_f_|. As shown in Figure 3D, the sensitivity increases as the downstream thermistor is positioned closer to the heater, because S_d_ more effectively senses the temperature field affected by blood flow convection. However, placing S_u_ sufficiently far upstream reduces the direct influence of the heater and provides a relatively stable upstream reference temperature. The sensitivity analysis located a point-based optimum at *d*_S*u*_ = 6.3 mm and *d*_S*d*_ = 2.0 mm, with a maximum sensitivity of approximately 13.45 °C/(m/s). The search range started at 2 mm, corresponding to the heater radius, because candidate sensor positions inside the heater footprint were excluded from the layout optimization. The point-based optimum cannot be implemented directly because each thermistor has a finite radius of 0.5 mm and must be offset from the 2.0 mm heater edge to avoid direct thermal contact with the heater. The downstream distance was therefore set to *d*_S*d*_ = 2.7 mm, which leaves a 0.2 mm clearance between the heater edge and the thermistor edge of S_d_. The upstream distance was relaxed to *d*_S*u*_ = 6.0 mm to accommodate the sensor footprint and component spacing. At the selected sensor-center locations, the point-based sensitivity is 10.4 °C/(m/s), indicating that the configuration remained within the high-sensitivity region identified under the specified optimization conditions. In the implemented sensor model, the temperatures were averaged over the 0.5 mm radius thermistor areas.

**Figure 4:**
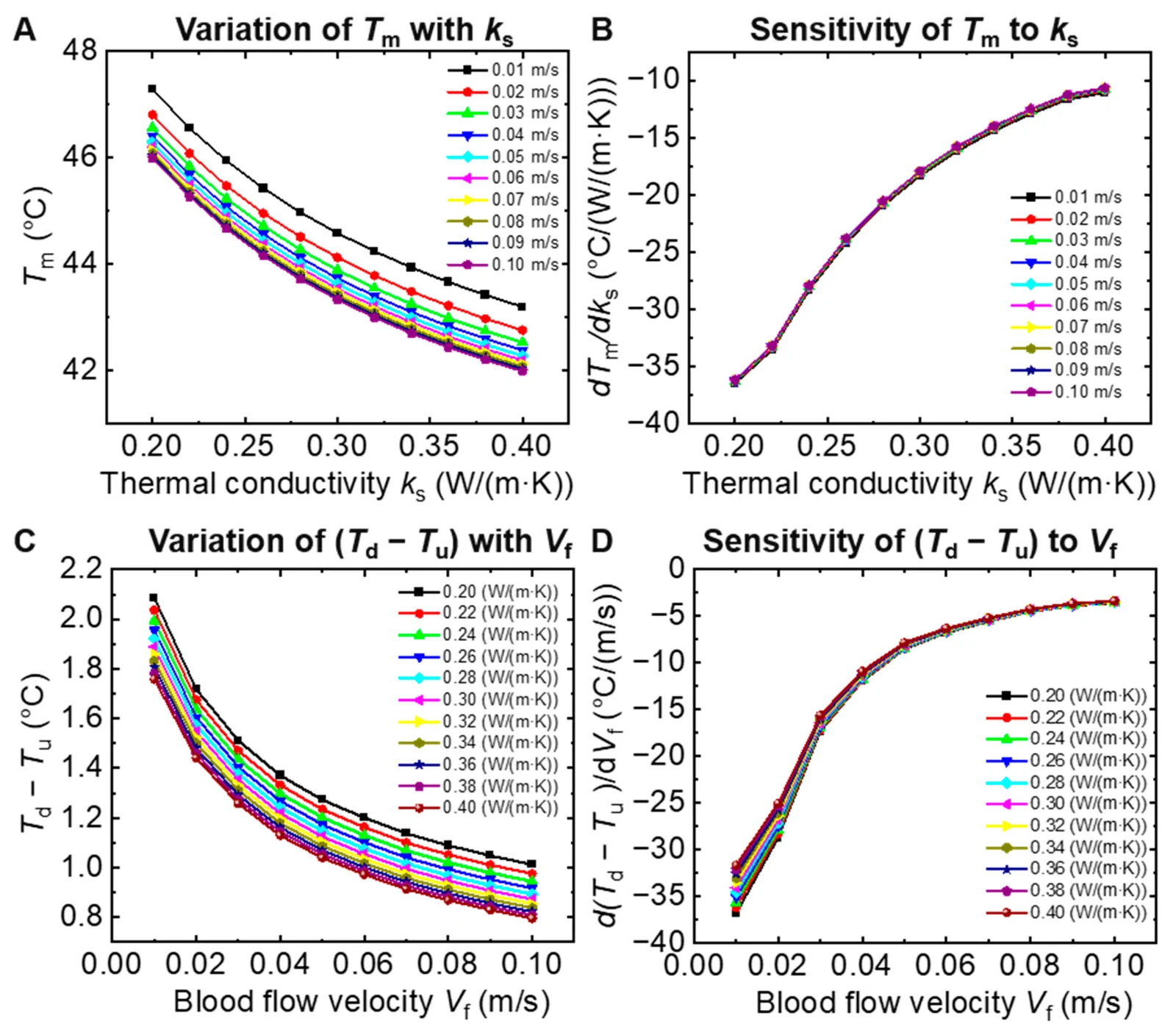
Thermal sensor response and sensitivity to tissue thermal conductivity *k*_s_ and blood flow velocity *V*_f_. (**A**) Variation in middle-sensor temperature *T*_m_ with *k*_s_ for a series of *V*_f_. (**B**) Corresponding sensitivity d*T*_m_/d*k*_s_. (**C**) Variation in differential temperature (*T*_d_ − *T*_u_) with *V*_f_ for a series of *k*_s_. (**D**) Corresponding sensitivity d(*T*_d_ − *T*_u_)/d*V*_f_.

Figure 4 shows the FEA results of temperature responses of the three thermistors for various *k*_s_ and *V*_f_. The middle-sensor temperature *T*_m_ decreases monotonically as *k*_s_ increases from 0.20 to 0.40 W/(m·K), as shown in Figure 4A. A larger *k*_s_ reduces the vertical and lateral conductive resistances beneath the heater, allowing the input heat to spread through a larger tissue volume more efficiently. Increasing *V*_f_ also lowers *T*_m_, but it produces a smaller and nearly parallel shift in the temperature curves, with negligible influence on the curve slope. Figure 4B analyzes the sensitivity of *T*_m_ to *k*_s_ and shows that d*T*_m_/d*k*_s_ is negative and changes from −36 to −11 °C/(W/(m·K)). As *k*_s_ increases, the sensitivity magnitude decreases because the same increment in *k*_s_ produces a progressively smaller fractional reduction in conductive resistance. The sensitivity curves for different *V*_f_ nearly overlap, meaning the shape and slope of the *T*_m_ response are affected mainly by *k*_s_, which makes the heater-centered thermistor suitable for determining tissue thermal conductivity. The differential temperature *T*_d_ − T_u_ reflects blood velocity, decreasing from 1.8~2.1 °C at *V*_f_ = 0.01 m/s to 0.8~1.0 °C at *V*_f_ = 0.10 m/s in Figure 4C. At low flow, the blood cannot remove heat effectively and the downstream region near the heater is heated more strongly. As *V*_f_ increases, enhanced convection removes heat and reduces *T*_d_. The magnitude of d(*T*_d_ − *T*_u_)/d*V*_f_ decreases from 32~37 to 3~4 °C/(m/s) as blood velocity increases in Figure 4D, showing blood convection saturation while conduction through the tissue becomes dominant. The overlapped curves for different *k*_s_ confirm that *T*_d_ − *T*_u_ is predominantly flow sensitive.

**Figure 5:**
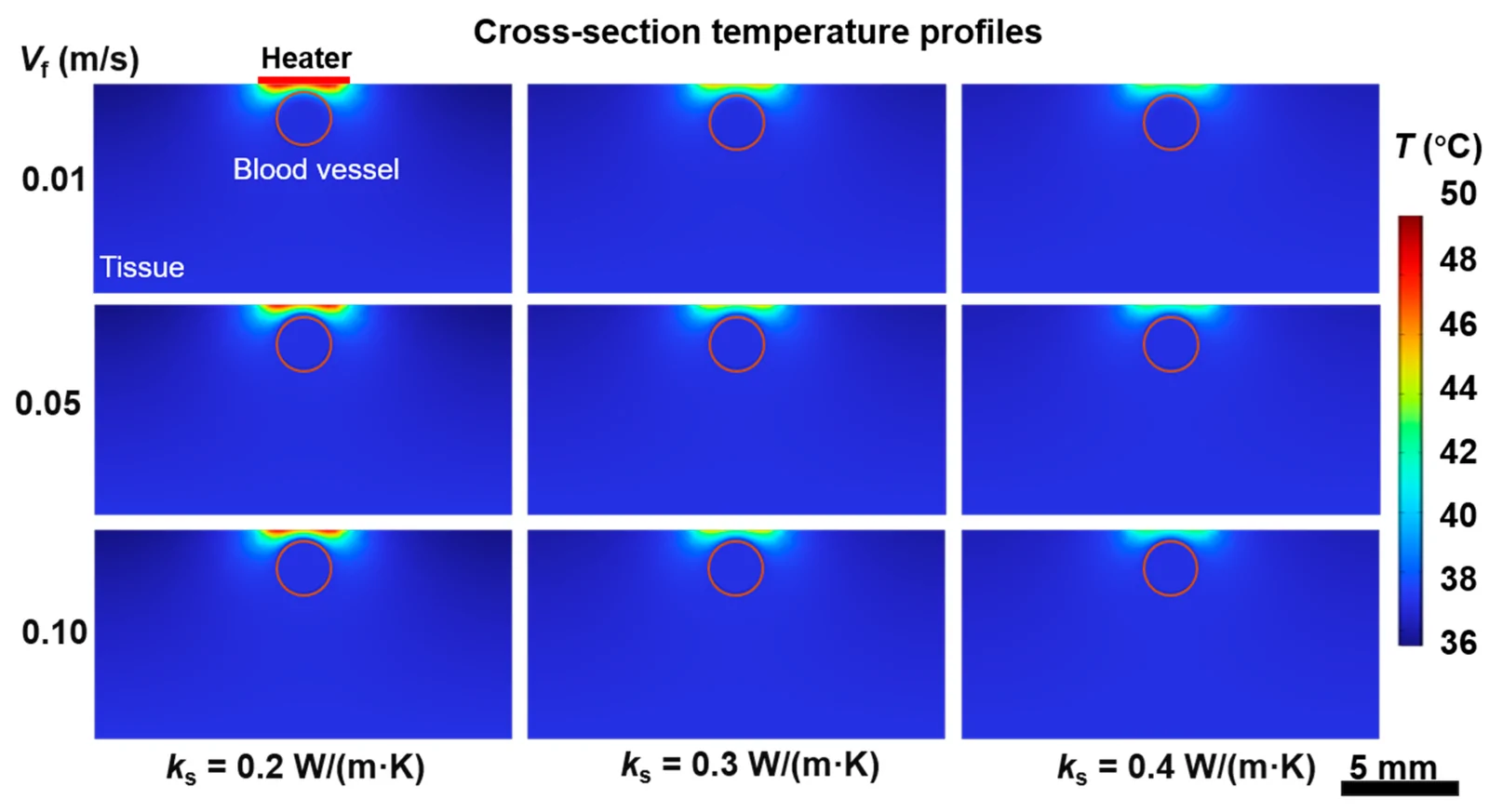
Temperature profiles in the cross-section view of the thermal sensor on tissue for selected *k*_s_ and *V*_f_. Temperature distributions are shown for tissue thermal conductivities *k*_s_ = 0.2 W/(m⋅K), 0.3 W/(m⋅K), 0.4 W/(m⋅K) and blood velocities *V*_f_ = 0.01 m/s, 0.05 m/s, 0.10 m/s.

Figure 5 shows cross-sectional temperature fields for selected *k*_s_ and *V*_f_. In every case, the maximum temperature occurs directly beneath the heater, and the temperature decreases rapidly at depth. The vessel remains close to the 37 °C inlet temperature and acts as a heat sink beneath the heated surface. At *k*_s_ = 0.20 W/(m·K), heat remains confined near the heater, producing the highest local temperature. Increasing *k*_s_ to 0.40 W/(m·K) lowers the maximum temperature and diffuses heat over a wider tissue region because both vertical and lateral conduction are enhanced. The effect of *V*_f_ is less obvious in the cross-sectional thermal profile than the effect of *k*_s_, because blood convection transports energy primarily along the vessel axis, which is normal to the displayed plane. The results demonstrate that the heater-centered temperature reflects a strong conductivity response, and a longitudinal upstream–downstream measurement is needed to characterize the flow response.

**Figure 6:**
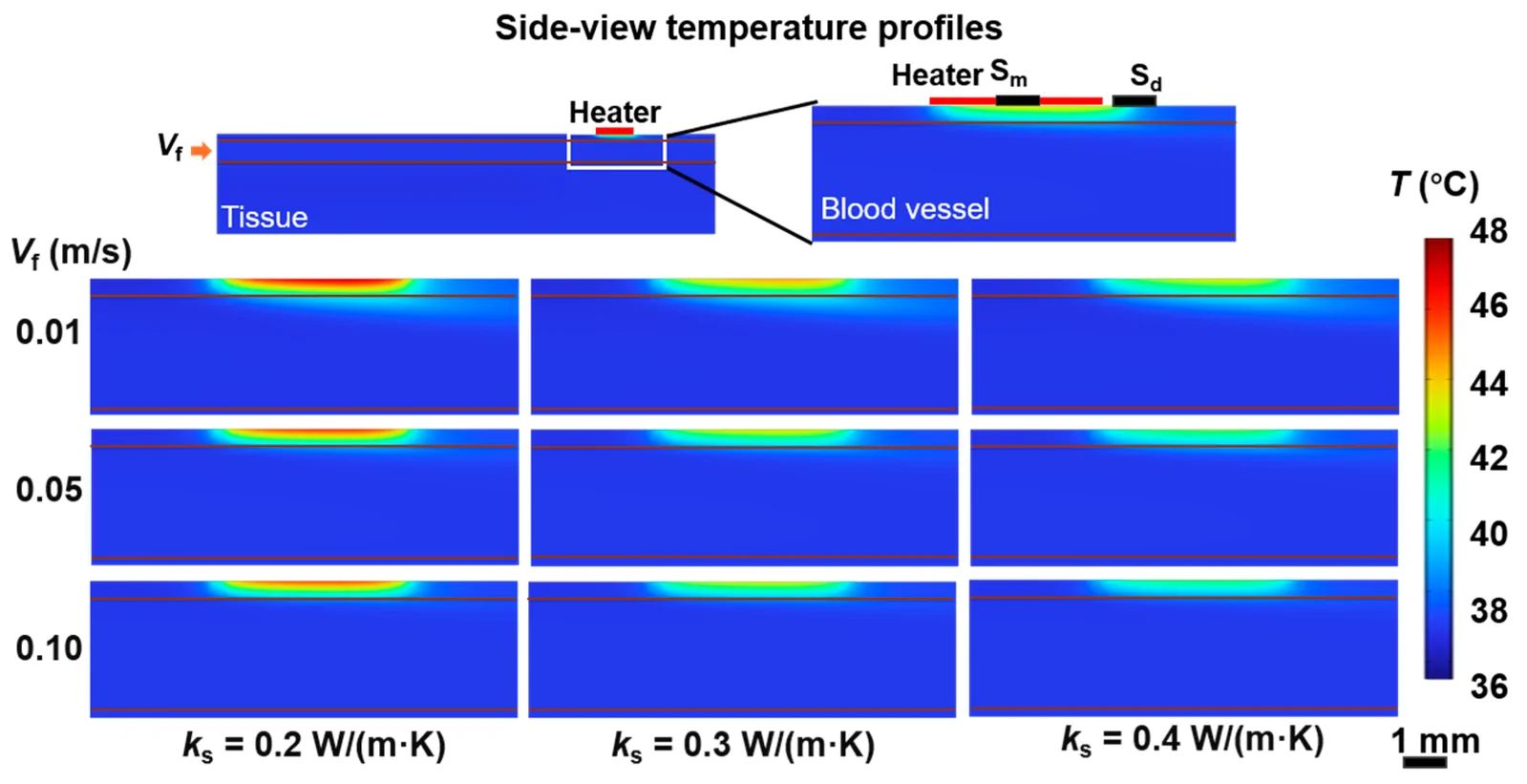
Zoomed-in side-view temperature profiles of the thermal sensor for selected *k*_s_ and *V*_f_. Temperature distributions are shown for tissue thermal conductivities *k*_s_ = 0.2 W/(m⋅K), 0.3 W/(m⋅K), 0.4 W/(m⋅K) and blood velocity *V*_f_ = 0.01 m/s, 0.05 m/s, 0.10 m/s. The solid lines indicate the blood vessel walls; the white box marks the zoomed-in region.

Figure 6 shows the temperature fields along the blood flow direction and the thermal wake that does not appear in the cross-sectional view. At low *V*_f_ of 0.01 m/s, weak blood convection reduces heat transfer and causes high temperature near the heater region. Increasing *V*_f_ to 0.05 and 0.10 m/s increases convective heat transfer, lowers the downstream tissue temperature, and shortens the region affected by the heater. The strongest velocity-dependent temperature change therefore occurs between the heater and *S*_d_, rather than at the upstream location. Increasing *k*_s_ lowers the surface maximum temperature for every velocity and conducts heat farther into the tissue. This conductivity effect changes the magnitude of the local temperature field but does not change the flow-induced directionality. The side-view fields therefore show that the velocity dependence is confined to the downstream region, while the conductivity dependence acts over the entire heated area.

**Figure 7:**
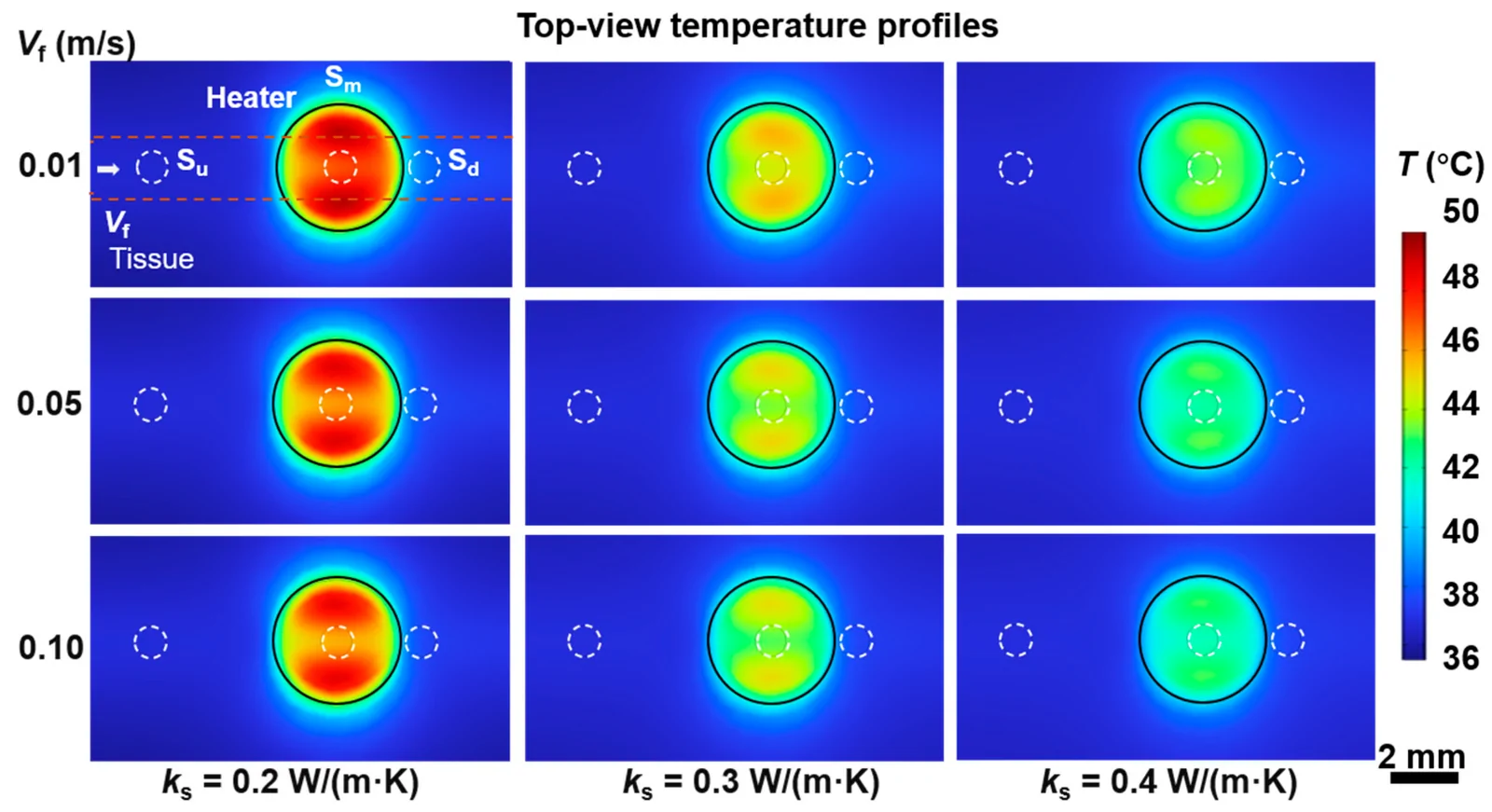
Top-view surface temperature profiles for selected *k*_s_ and *V*_f_. Surface temperature distributions are shown for tissue thermal conductivities *k*_s_ = 0.2 W/(m⋅K), 0.3 W/(m⋅K), 0.4 W/(m⋅K) and blood velocities *V*_f_ = 0.01 m/s, 0.05 m/s, 0.10 m/s. The dashed red lines outline the blood vessel. The dashed white circles outline the three thermistors. The solid black circle outlines the heater.

Figure 7 shows the corresponding skin surface temperature profiles. At *k*_s_ = 0.20 W/(m·K), the heater region has the highest temperature and the hot spot is confined within the heater, due to weak thermal conduction. As *k*_s_ increases, the peak temperature decreases and the surface temperature gradient becomes smoother because heat spreads laterally away from the heater efficiently. The temperature change is directly measured by S_m_, which is located near the center of the conductivity-dependent hot spot. Blood flow mainly changes the temperature field on the downstream side of the heater. At low *V*_f_, heat remains in the vessel-coupled region and produces a higher temperature near S_d_. At high *V*_f_, the blood dilutes more heat, reducing the downstream surface temperature and the difference in *T*_d_ − *T*_u_. The temperature at S_u_ has negligible change because the thermistor is located far upstream of the heater. In summary, the temperature profiles from FEA simulations show that *k*_s_ affects the magnitude and spatial spreading of the surface heater significantly, and *V*_f_ affects the longitudinal asymmetry greatly, forming the physical foundation to apply *T*_m_ and *T*_d_ − *T*_u_ for simultaneous measurements of tissue thermal conductivity and blood velocity.

The distinct dependence of the three thermistor temperatures on *k*_s_ and *V*_f_, as established by the above FEA results, provides the basis for data-driven parameter estimation by an ML algorithm. Each FEA case associates a temperature triplet [*T*_u_, *T*_m_, *T*_d_] with a known parameter pair [*k*_s_, *V*_f_]. Supervised regression utilizes these paired data to learn a temperature-to-parameter mapping by minimizing the error between model predictions and FEA ground truth. Once trained, this mapping enables *k*_s_ and *V*_f_ to be estimated from thermistor temperatures measured by the thermal sensor. Seven candidate regression models were evaluated using shuffled five-fold cross-validation. Third-order polynomial ridge regression produced the lowest mean normalized root-mean-square error and was therefore selected. During model training, the three temperature inputs were standardized and transformed into third-order polynomial features containing nonlinear and interaction terms. The coefficients were estimated by ridge regression, which applies L2 regularization. L2 regularization adds a penalty based on the sum of the squared regression coefficients to the model loss, thereby discouraging excessively large coefficients, reducing overfitting, and improving model stability. For each prediction, the measured temperature triplet was standardized using the training-set mean and standard deviation, transformed into the same polynomial feature space, and passed to the fitted regression model to obtain predictions of *k*_s_ and *V*_f_. Candidate-model settings and cross-validation performance are summarized in Supplementary Table S2.

The generalization performance of the selected model was further evaluated using 30 independently simulated off-grid FEA cases that were not included in model training or selection. None of these parameter combinations coincided with the original full-factorial grid, and the fitted model was kept fixed, without retraining or model reselection, throughout the validation. The selected model achieved *R*^2^ values of 0.99976 for *k*_s_ and 0.99424 for *V*_f_ on the 30 independent off-grid FEA cases, demonstrating high predictive accuracy for previously unseen parameter combinations within the investigated domain. Complete validation metrics and bootstrap 95% intervals are provided in Supplementary Table S3.

**Figure 8:**
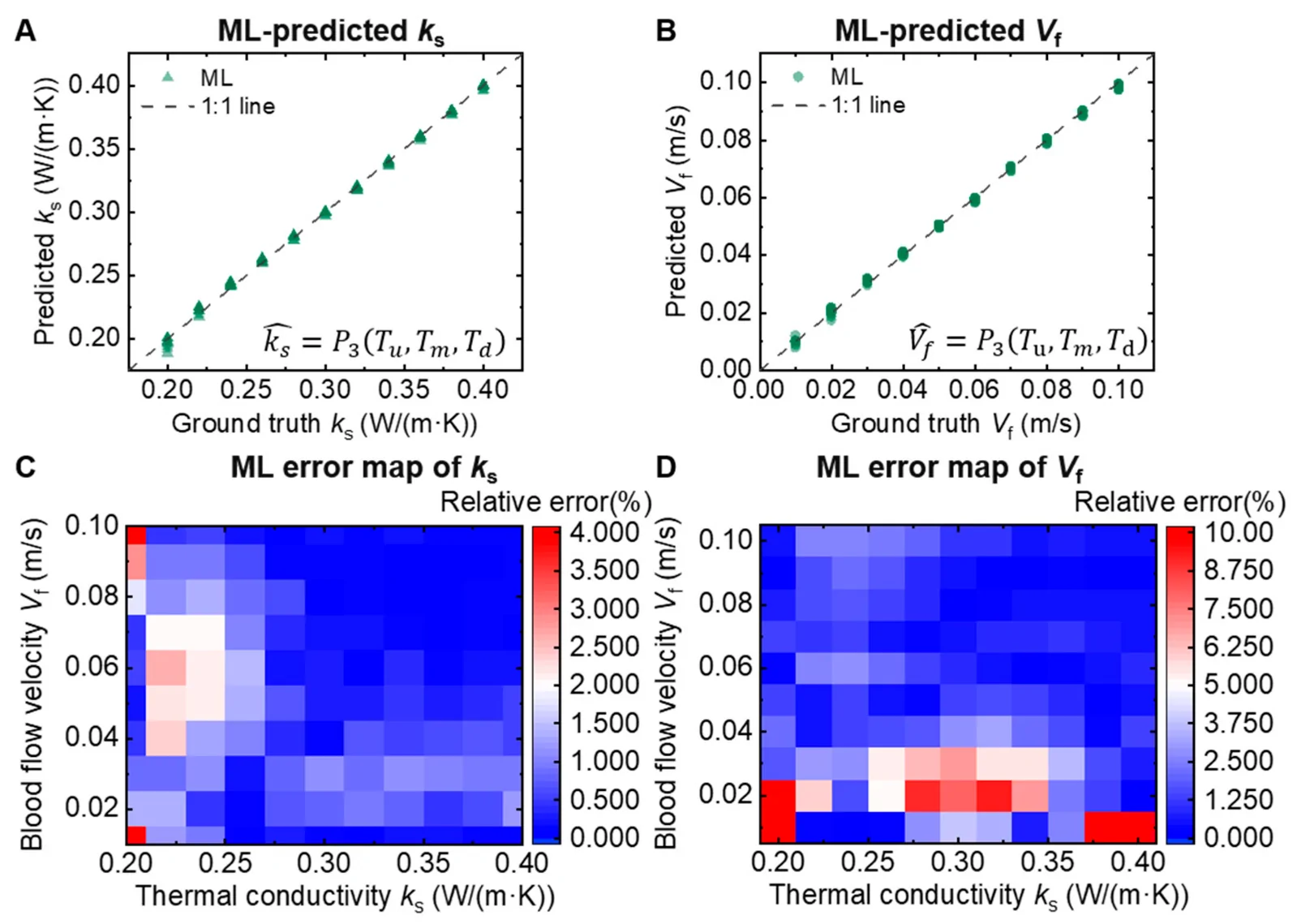
Machine learning prediction results. (**A**) Tissue thermal conductivity *k*_s_ predicted by machine learning (ML) and ground-truth FEA modeling. (**B**) Blood flow velocity *V*_f_ predicted by ML and ground-truth FEA modeling. (**C**) Relative prediction error map of ML-predicted *k*_s_ with respect to the FEA reference. (**D**) Relative prediction error map of ML-predicted *V*_f_ with respect to the FEA reference. P3 denotes a 3rd-order polynomial regression mapping the three sensor temperatures to the inverse-predicted *k*_s_ and *V*_f_. Note that the color bar scales for *k*_s_ and *V*_f_ are capped at 4.0% and 10.0%, respectively, to optimize visual contrast across the primary domain; these caps are display limits and not the maximum prediction errors.

Figure 8 shows the ML predictions of *k*_s_ and *V*_f_ from *T*_u_, *T*_m_, and *T*_d_. The predicted *k*_s_ values at various blood velocities match well with the ground-truth FEA simulations, with only slight deviations at low thermal conductivities in Figure 8A. The ML-predicted *V*_f_ values are also validated by FEA simulations in Figure 8B. These results show that the third-order polynomial model is able to accurately predict the coupled *k*_s_ and *V*_f_ from the three temperatures collected from one thermal sensor measurement rather than requiring separate measurements for each parameter. The relative prediction error maps in Figure 8C,D show the distributions of ML prediction accuracy across various combinations of *k*_s_ and *V*_f_. Prediction errors in *k*_s_ are low over most combinations and are relatively high near the conductivity and velocity boundaries, where polynomial interpolation in ML algorithm is supported by fewer neighboring cases. Prediction errors in *V*_f_ are also low through the interior of the domain but become larger at low velocity. Because relative prediction errors are normalized by *V*_f_, a similar absolute deviation produces a larger percentage error at *V*_f_ = 0.01 m/s. The largest relative prediction errors of the ML model, 5.58% for *k*_s_ and 21.92% for *V*_f_, both occur at the same corner case of *k*_s_ = 0.20 W/(m·K) and *V*_f_ = 0.01 m/s, where the prediction lies simultaneously at the lower bound of both parameter ranges and the percentage error is amplified by the small denominator. Excluding such isolated corner cases, the relative prediction error remains below 4% for *k*_s_ predictions and below 10% for *V*_f_ predictions. These results demonstrate that the ML model can simultaneously predict the two coupled parameters from the three thermistor temperatures with good agreement with the FEA reference over the interior of the parameter space.

**Figure 9:**
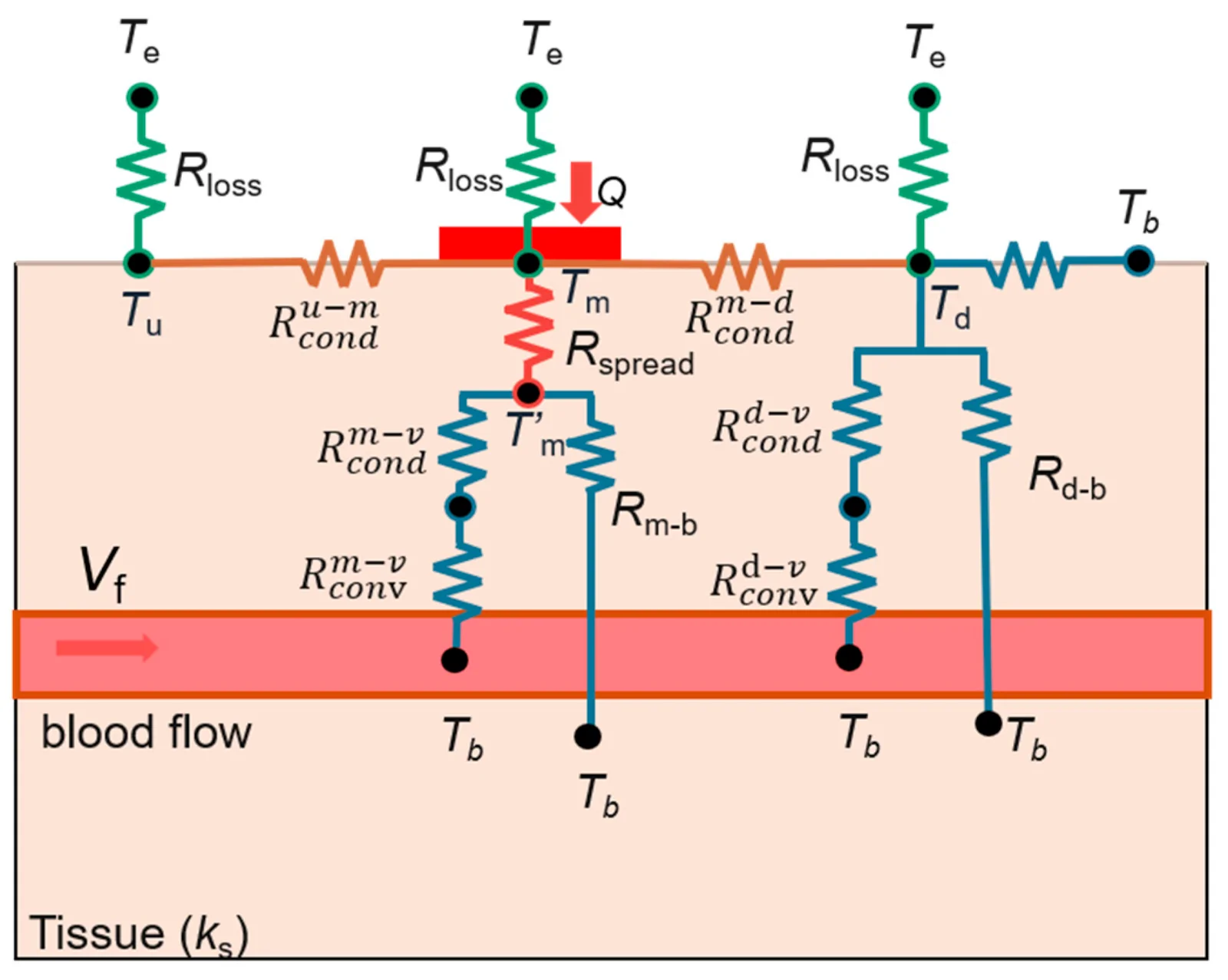
Reduced-order model (ROM) of the thermal-resistance network for heat transfer analysis from the thermal sensor to blood flow, tissue, and environment.

To further investigate the conjugate heat transfer mechanism in tissue and blood flow, the thermal-resistance network is built to describe heat conduction from the thermal sensor to the tissue, blood convection, and environment heat loss in Figure 9. The three thermistor locations are represented by surface nodes u, m, and d. The heater supplies *Q =* 0.05 W at node m, and lateral conductive resistances connect the heater region to the upstream and downstream regions. Each surface node also transfers heat to the ambient environment through air natural-convection loss resistance. At nodes m and d, heat is divided between two parallel paths of tissue and vessel blood. In the vessel-coupled path, heat first conducts through the tissue layer above the vessel and is then transferred by blood convection. In the bypass path, additional heat conducts around the vessel and dissipates into deeper tissue. At node m, *R*_spread_ connects the measured heater temperature *T*_m_ to the effective subsurface temperature *T’_m_.* This resistance accounts for the additional temperature rise created when heat spreads from the finite heater area into a larger tissue volume. The network represents the major conductivity dependence by conductive and spreading resistances and the major flow dependence by the blood convective resistances. Increasing *k*_s_ decreases the lateral, vertical, bypass, and spreading resistances, reducing *T*_m_ significantly. Increasing *V*_f_ increases the Nusselt number and heat transfer coefficient, thereby changing the downstream temperature *T*_d_*. T*_u_ is a measured reference, while *T*_m_ and *T*_d_ reflect the conduction- and flow-dependent responses. The thermal-resistance network clearly demonstrates different heat transfer pathways in the complex thermal-fluid scenario and establishes the foundation for ROM heat transfer analysis.

**Figure 10:**
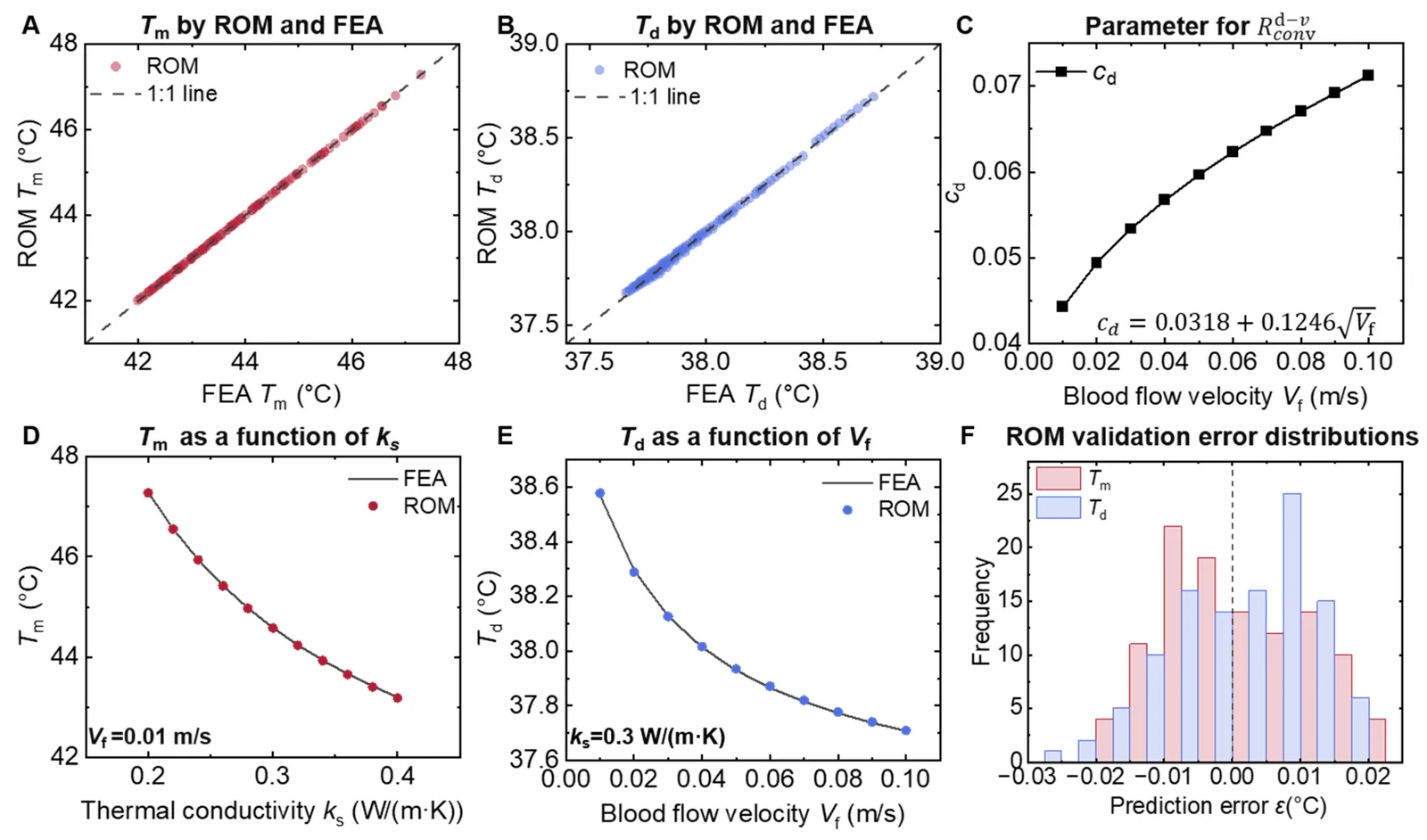
Validation of the reduced-order model (ROM) by the FEA results. (**A**) ROM versus FEA for *T*_m_. (**B**) ROM versus FEA for *T*_d_. (**C**) Calibration of the downstream convective resistance $R_{conv}^{d-v}$ as a nonlinear function of blood velocity *V*_f_. (**D**) Comparison of *T*_m_ calculated by ROM and FEA as a function of *k_s_* at *V*_f_ = 0.01 m/s. (**E**) Comparison of *T*_d_ calculated by ROM and FEA as a function of *V*_f_ at *k_s_ =* 0.3 W/(m·K). (**F**) ROM calculation error distributions for *T*_m_ and *T*_d_, compared with ground-truth FEA. The vertical dashed line indicates zero prediction error. *T*_u_ is lumped isothermal temperature with negligible change by less than 0.3 °C.

Figure 10 compares the ROM-calculated temperatures with FEA simulations across full *k*_s_ and *V*_f_ ranges. The detailed ROM formulation and calibration procedure are provided in Supplementary Section S1. *T*_m_ derived by ROM closely matches the FEA results in Figure 10A, and similarly good agreement is observed for *T*_d_ in Figure 10B. Figure 10C shows the calibrated parameter *c*_d_ for the downstream convective resistance at node d. *c*_d_ is a function of *V*_f_^1/2^ and increases with velocity, indicating that downstream convective heat transfer strengthens as *V*_f_ increases. This nonlinear dependence is associated with the development of the local boundary layer and blood convective transport near the downstream sensor. Figure 10D further shows that the ROM predicts the nonlinear decrease in *T*_m_ with *k*_s_ at *V*_f_ = 0.01 m/s as an example. Figure 10E shows *T*_d_ decreases with *V*_f_ at *k*_s_ = 0.30 W/(m·K), calculated by both ROM and FEA methods. Figure 10F summarizes the ROM calculation error of *T*_m_ and *T*_d_ compared with FEA results. The calculation errors are narrowly distributed around 0 °C, with magnitudes within only a few hundredths of a degree Celsius. Figure 10 demonstrates that the ROM is sufficiently accurate as a forward model, which provides the basis for using it to determine *k*_s_ and *V*_f_ from thermistor temperature measurements.

**Figure 11:**
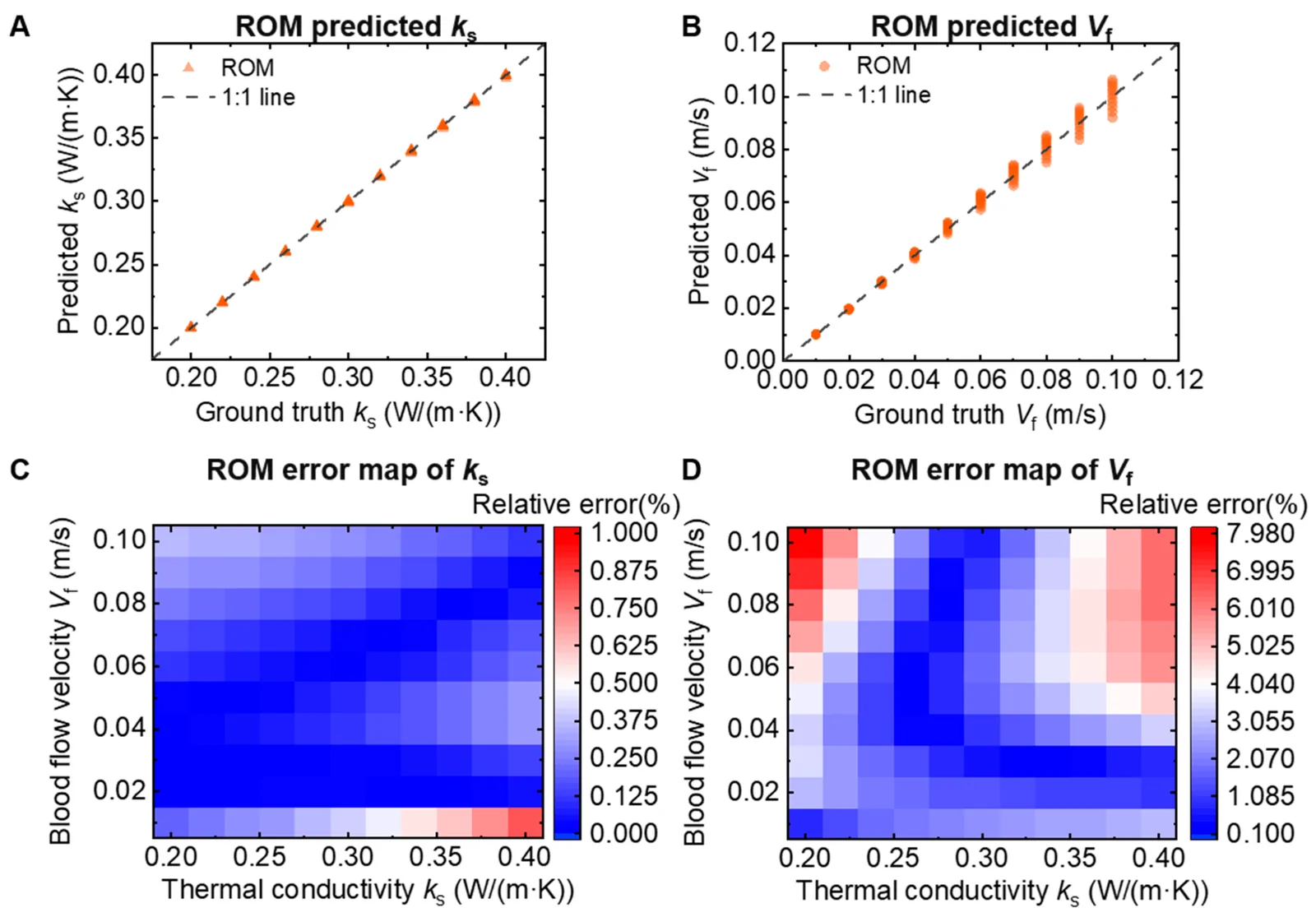
Reduced-order model (ROM) prediction results. (**A**) Tissue thermal conductivity *k*_s_ predicted by ROM and ground-truth FEA modeling. (**B**) Blood flow velocity *V*_f_ predicted by ROM and ground-truth FEA modeling. (**C**) Relative prediction error map of ROM-predicted *k*_s_ with respect to the FEA reference. (**D**) Relative prediction error map of ROM-predicted *V*_f_ with respect to the FEA reference.

Figure 11 shows the ROM predictions of *k*_s_ and *V*_f_ derived from the three thermistor temperatures *T*_u_, *T*_m_, and *T*_d_. Figure 11A,B compare the ROM-predicted *k*_s_ and *V*_f_ with the FEA ground-truth values. The predicted *k*_s_ values closely follow the 1:1 line throughout the investigated range, demonstrating that the ROM accurately recovers tissue thermal conductivity from the temperature measurements. The predicted *V*_f_ values also show good overall agreement with the FEA reference, although the dispersion around the 1:1 line becomes more obvious at higher flow velocities. Figure 11C,D show the relative prediction error distributions of the ROM predictions across the *k*_s_ − V_f_ parameter space. The relative prediction error in *k*_s_ remains below 1% throughout the investigated domain, indicating consistently high prediction accuracy with limited dependence on blood flow velocity. The relative prediction error in *V*_f_ remains low over most of the parameter space but increases near the parameter boundaries and at higher flow velocities, reaching up to 8%. The increased *V*_f_ prediction error at high flow velocities is consistent with the reduction in thermal sensitivity when convective heat transfer becomes strong and thermal convection resistance is less dominant than tissue thermal conduction. At high flow velocities, a given change in *V*_f_ produces a smaller temperature response, making the inferred *V*_f_ more susceptible to small temperature errors. These results demonstrate that the ROM can use the three thermistor temperatures to simultaneously predict *k*_s_ and *V*_f_ with high accuracy. In addition to providing a computationally efficient prediction method, the physics-based ROM explicitly relates the measured thermal sensor temperatures to conductive heat spreading in the tissue and convective heat transfer by blood flow.

**Figure 12:**
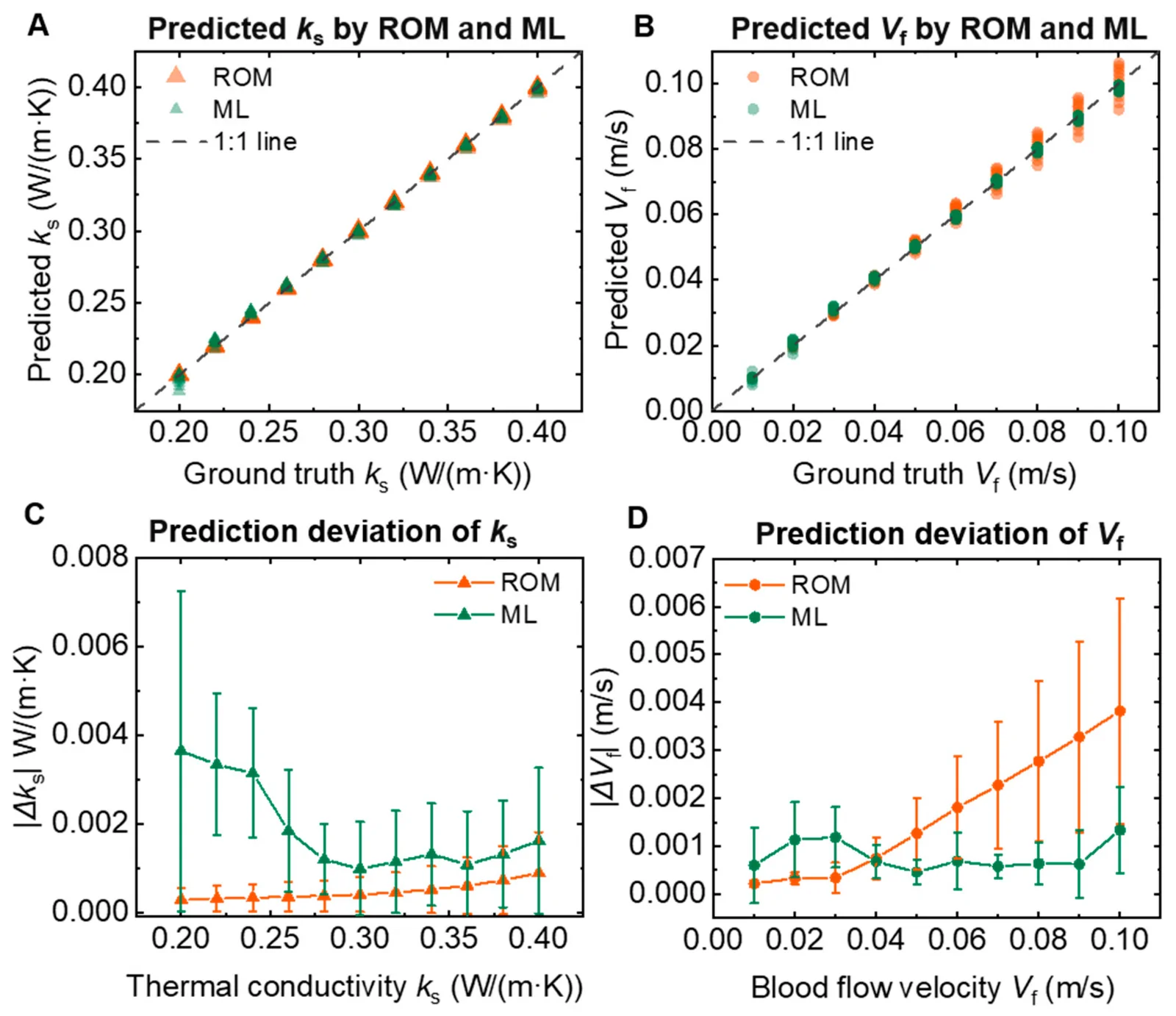
Comparison between the reduced-order model (ROM) and machine learning (ML) predictions. (**A**) Comparison of ROM- and ML-predicted *k*_s_ validated by the ground truth. (**B**) Comparison of ROM- and ML-predicted *V*_f_ validated by the ground truth. (**C**) Absolute prediction deviation ∆*k*_s_ by ROM and ML for tissue thermal conductivity. (**D**) Absolute prediction deviation ∆*V*_f_ by ROM and ML for blood flow velocity.

Figure 12 compares the prediction performance of the ROM and ML models and provides an overall evaluation of the proposed thermal sensor design to measure tissue thermal conductivity and blood velocity. Figure 12A,B show the *k*_s_ and *V*_f_ predicted by the two models and compare the predictions with FEA ground-truth values. Both models accurately predict *k*_s_ and *V*_f_ over the investigated range, with the prediction points following the 1:1 line. However, differences between the two methods become more evident from the absolute prediction deviations shown in Figure 12C,D. As shown in Figure 12C, the ROM has smaller prediction errors in *k*_s_ than the ML model across the investigated conductivity range. The improvement is particularly significant near the lower *k*_s_ boundary, where the ML model shows greater prediction errors. This result indicates that the physical constraints incorporated into the ROM provide robust prediction of tissue thermal conductivity because the ROM is less susceptible to the data-driven boundary effects that degrade the ML predictions near the edges of the training parameter space. For *V*_f_ prediction, Figure 12D shows that the two models exhibit different prediction error characteristics. The ROM provides small deviations at low flow velocities, but the deviation gradually increases as *V*_f_ increases. In contrast, the ML model generally maintains smaller deviations over the intermediate-to-high flow range, although it shows greater deviations in low flow conditions. The increasing ROM deviation at high *V*_f_ is associated with the reduced temperature sensitivity under conduction resistance dominant conditions. The ML model more flexibly captures the nonlinear temperature–flow relationship, resulting in improved *V*_f_ prediction at higher velocities.

More importantly, the agreement between the two fundamentally different prediction frameworks supports the feasibility of the proposed thermal sensor configuration. The ROM represents the sensor response using physically defined conductive and convective thermal resistances, whereas the ML model learns the temperature-to-parameter relationship directly from the FEA dataset. Despite these different representations, both models successfully distinguish the effects of tissue thermal conductivity and blood flow velocity from a single set of three thermistor measurements. Because the two models employ different physical representations and modeling assumptions, their agreement indicates that the simultaneous prediction capability arises from the complementary thermal information captured by the thermal sensor layout rather than from a specific prediction algorithm. The ROM provides physical interpretability and higher accuracy in predicting *k*_s_, whereas the ML model provides greater flexibility in capturing the nonlinear flow response.

## 4. Discussion

The design and predicted performance of the proposed skin-interfaced thermal sensor are evaluated as a numerical proof of concept under simplifying assumptions. These assumptions enable the effects of tissue thermal conductivity and blood flow velocity on the sensor response to be systematically isolated, while defining the current applicability of the sensor design and its associated prediction models.

The tissue is represented by a single effective thermal conductivity because the surface thermistors respond to the integrated thermal behavior of the heat-affected tissue volume rather than to individual skin layers. The effective conductivity therefore lumps the multilayer structure into a single equivalent property of the heat-affected region, which is both the quantity the sensor interrogates and a single identifiable unknown that keeps the inverse problem well-posed. Vascular geometry is represented by a single superficial vein of fixed diameter and depth, with both values selected according to anatomical considerations. This fixes a well-defined baseline for the present analysis; because the framework is parameterized by the vessel geometry, it can be extended to vessels of other diameters, depths, and geometrical features by rebuilding the model with the corresponding parameters. Natural convection at the skin surface is treated with a prescribed boundary condition, a representative value for quiescent air easily controlled by sensor encapsulation. In the ROM, the corresponding surface convective resistance is large relative to the conductive and blood convective resistances near the heater, indicating that surface heat loss is a secondary pathway under the modeled conditions.

The ROM offers a complementary and physically transparent representation of the same heat transfer process by resolving the thermal pathways from the heater to the thermistors into a network of resistances associated with tissue conduction, blood flow convection, lateral heat spreading, and heat exchange at the skin surface. This decomposition clarifies why the proposed sensor configuration contains distinguishable information for estimating the two unknown parameters. The middle-thermistor temperature is primarily influenced by the tissue-conduction and heat-spreading pathways and is therefore predominantly sensitive to *k*_s_, whereas the downstream temperature relative to the upstream reference is more strongly influenced by the flow-dependent convective pathway and is therefore predominantly sensitive to *V*_f_. The simultaneous estimation of *k*_s_ and *V*_f_ consequently arises from the different pathway sensitivities captured by the thermistor arrangement rather than from numerical fitting alone. A small number of network parameters representing three-dimensional geometric, spreading, and localized convective effects that do not admit simple closed-form descriptions are calibrated using the FEA data. The ability of the resulting network to reproduce the FEA temperature response across the investigated parameter domain with only these coefficients indicates that the dominant heat transfer behavior is captured by the network structure, while the calibrated parameters account for geometric details omitted from the reduced-order representation. Although the ROM and ML models share the same FEA-defined physical domain, they represent the inverse relationship differently: the ML model learns the temperature-to-parameter mapping from data, whereas the ROM imposes a physically structured thermal-resistance network. Their agreement therefore provides complementary evidence that the sensor layout contains sufficient and distinguishable thermal information for the simultaneous estimation of *k*_s_ and *V*_f_.

The next step in translating the numerical sensor design into physical measurement is to fabricate and evaluate a wearable prototype under controlled thermal boundary conditions. The sensing region will be implemented on a flexible printed circuit board with a total thickness below 0.2 mm, allowing it to conform closely to the skin, and will integrate miniature thermistors, a resistive heater, and a wireless data-acquisition module. During sensor encapsulation, a sealed air dome will be incorporated into the rigid sensor enclosure to suppress ambient airflow and maintain a stable air layer above the sensing region for well-controlled natural convection. Experimental measurements will require consideration of the skin–sensor thermal contact resistance, which may introduce an interfacial temperature drop and affect the inferred parameters. Its influence will be evaluated by controlling the interface material and mounting pressure, together with the effects of sensor positioning, component tolerances, heater-power stability, and motion. Initial validation will be conducted using tissue-mimicking phantoms with independently characterized thermal conductivity and controlled flow velocity. These experiments will support refinement of the inverse model and provide the basis for a measurement-uncertainty evaluation following the GUM framework.

## 5. Conclusions

This study presented and numerically validated a skin-interfaced thermal sensor for the simultaneous prediction of *k*_s_ and *V*_f_ from a single heating and measurement scenario. Both the data-driven ML model and the physics-based ROM accurately predicted *k*_s_ and *V*_f_ over the investigated ranges of 0.20~0.40 W/(m·K) and 0.01~0.10 m/s, respectively, with relative prediction errors typically below 4% and 10% using the ML model and below 1% and 8% using the ROM. Although the two frameworks represented the heat transfer process differently, their consistent predictions indicated that the simultaneous prediction capability originated from the proposed thermal sensor configuration rather than from a specific prediction algorithm. A single device therefore returns complementary information on local skin condition and vascular flow status, which is not accessible from either parameter alone. This work established the theory and computational basis for implementing the proposed sensing and prediction framework in a wearable thermal sensor.

## Data Availability

The original contributions presented in this study are included in the article/Supplementary Material. Further inquiries can be directed to the corresponding author.

## Supplementary Materials

The following supporting information can be downloaded at: https://www.mdpi.com/article/10.3390/mi17091093/s1. Figure S1: FEA-based calibration of lateral conductive thermal resistance; Figure S2: Effective conductive areas for bypass resistance estimation; Figure S3: Temperature distribution at node u; Table S1: Geometric, thermophysical, boundary, and calibrated parameters used in the FEA and reduced-order thermal-resistance model; Table S2: Five-fold cross-validation performance of the candidate machine-learning models. Table S3: Independent off-grid validation performance of the selected machine-learning inverse model.
