# Supplementary material for: Numerical Investigation of a Skin-Interfaced Thermal Sensor for Joint Estimation of Tissue Thermal Conductivity and Blood Velocity
Source: Micromachines (Basel). 2026 Sep 17;17(9):1093. doi: 10.3390/mi17091093 (PMC13609165; doi:10.3390/mi17091093)
Supplement: Supplementary file 1 [file micromachines-17-01093-s001.zip › micromachines-4525483-supplementary.pdf]

Supplementary Information

S1.Thermal resistance network analysis

This section describes the formulation and FEA-based calibration of the thermal-resistance network introduced in Figure 9. Figure S3 shows that across the complete parameter sweep, T_u ranged from 36.63 to 36.86 °C, corresponding to a total variation of approximately 0.23 °C. This weak dependence on k_s and V_f supported representing the upstream thermistor as a lumped temperature node in the ROM. At node m, the finite heater footprint produces three-dimensional heat spreading, causing the measured temperature T_m to differ from the effective network temperature T'_m. This effect was represented by the spreading resistance R_{spread}. The effective middle-node temperature T'_m was first obtained from the network, and the measured temperature was reconstructed as

$$T_m = T'_m + QR_{\text{spread}};\ R_{\text{spread}} = \frac{C_{\text{spread}}}{k_s} \tag{S1}$$

where C_{spread} is a calibrated geometric coefficient. This form preserves the inverse dependence of spreading resistance on tissue thermal conductivity while avoiding an additional distributed temperature field in the ROM.

Lateral conduction between the surface nodes was represented by

$$R_{\text{cond},u-m} = \frac{L_u}{k_s A_{u-m}^{\text{lat}}};\ R_{\text{cond},m-d} = \frac{L_d}{k_s A_{m-d}^{\text{lat}}} \tag{S2}$$

The effective lateral areas were determined from FEA rather than from the geometric cross sections. For each separation plane j, the total lateral heat rate and the node-to-node temperature difference gave the effective resistance. The slope of the resistance plotted against $1/k_s$ was then used to obtain the effective area:

$$R_{\text{cond},j} = \frac{\Delta T_j}{\dot{Q}_j};\ S_j = \frac{dR_{\text{cond},j}}{d(1/k_s)} = \frac{L_j}{A_j^{\text{lat}}};\ A_j^{\text{lat}} = \frac{L_j}{S_j} \tag{S3}$$

The fitted slopes for the u−m and m−d planes were 2006.25 and 536.82 m^{-1}, respectively, corresponding to effective areas of 2.99 and 5.03 mm^2 (Figure S1). The larger downstream effective area reflects the stronger heat flow in the region immediately adjacent to the heater.

At nodes m and d heat was divided between a vessel-coupled branch and a parallel bypass branch into deep tissue. The vessel-coupled branch contains conduction across the tissue gap $\delta = h_v - r_v$ followed by blood convection, whereas the bypass branch represents heat conducted around the vessel:

$$R_{\text{cond},i\to v} = \frac{\delta}{k_s A_{w,i}};\ R_{i\to b} = \frac{L_{i,b}}{k_s A_{i,b}^{\text{eff}}} \tag{S4}$$

where i denotes node m or d. The thermally active cross section was identified from the FEA region above 37 °C and separated into the vessel-coupled area and the bypass area. The representative areas and conduction lengths are listed in Table S1. The most extensive thermal penetration occurred at high k_s and low V_f and remained within the vessel-center depth; therefore, both bypass lengths were set to 3 mm (Figure S2).

Blood flow was approximated as steady laminar flow in a circular tube. The vessel diameter, Reynolds number, and Prandtl number were

$$D_v = 2r_v; \mathrm{Re}_D = \frac{\rho_f V_f D_v}{\mu_f}; \mathrm{Pr} = \frac{\mu_f c_{p,f}}{k_f} \quad \text{(S5)}$$

The cumulative mean Nusselt number in the thermally developing region was calculated using the Hausen correlation:

$$\beta(x) = \left(\frac{D_v}{x}\right) \mathrm{Re}_D \mathrm{Pr}; \mathrm{Nu}_{\mathrm{avg}}(x) = 3.66 + \frac{0.0668\beta(x)}{1 + 0.04\beta(x)^{2/3}} \quad \text{(S6)}$$

The raw middle-node value was evaluated from the inlet to the middle location,

$$\mathrm{Nu}_{m,\mathrm{raw}} = \mathrm{Nu}_{\mathrm{avg}}(x_m) \quad \text{(S7)}$$

The downstream value was obtained as the length-weighted average over the downstream sensor segment:

$$\mathrm{Nu}_{d,\mathrm{raw}} = \frac{x_{d,2}\mathrm{Nu}_{\mathrm{avg}}(x_{d,2}) - x_{d,1}\mathrm{Nu}_{\mathrm{avg}}(x_{d,1})}{x_{d,2} - x_{d,1}} \quad \text{(S8)}$$

Because the one-dimensional tube correlation does not resolve the three-dimensional coupling between the heater, tissue, and vessel, FEA-calibrated correction factors were applied to the two local Nusselt numbers:

$$\mathrm{Nu}_{m,\mathrm{eff}} = c_m \mathrm{Nu}_{m,\mathrm{raw}}; \mathrm{Nu}_{d,\mathrm{eff}} = c_d \mathrm{Nu}_{d,\mathrm{raw}} \quad \text{(S9)}$$

$$c_d = c_{d,0} + c_{d,1}\sqrt{\frac{V_f}{V_0}}; V_0 = 1\ \mathrm{ms}^{-1} \quad \text{(S10)}$$

The constant middle-node factor accounts for the heater-centered coupling, whereas the velocity-dependent downstream factor captures the change in the local thermal wake with blood velocity. The reference velocity in Eq. (S10) keeps the fitted coefficients dimensionless. The local heat-transfer coefficient and convective resistance were then

$$h_i = \frac{k_f \mathrm{Nu}_{i,\mathrm{eff}}}{D_v}; R_{\mathrm{conv},i\to v} = \frac{1}{h_i A_{w,i}} \quad \text{(S11)}$$

and the complete vessel-coupled resistance was the series combination

$$R_{i\to v} = R_{\mathrm{cond},i\to v} + R_{\mathrm{conv},i\to v} \quad \text{(S12)}$$

Heat loss from each surface region to the ambient environment was represented by natural convection. All resistance branches were written as conductances for the nodal solution:

$$R_{\mathrm{loss},i} = \frac{1}{h_e A_i}; G_j = \frac{1}{R_j} \quad \text{(S13)}$$

At steady state, the energy balances at the effective middle node and the downstream node were

$$G_{u-m}(T'_m - T_u) + G_{m-d}(T'_m - T_d) + G_{m\to v}(T'_m - T_b) + G_{m\to b}(T'_m - T_b) + G_{\mathrm{loss},m}(T'_m - T_e) = Q \quad \text{(S14)}$$

$$G_{m-d}(T_d - T'_m) + G_{d\to v}(T_d - T_b) + G_{d\to b}(T_d - T_b) + G_{\mathrm{loss},d}(T_d - T_e) = 0 \quad \text{(S15)}$$

Equations (S14) and (S15) were solved simultaneously for T_m' and T_d and Eq. (S1) was subsequently used to calculate the thermistor temperature T_m. The geometric, thermophysical, boundary, and calibrated parameters are summarized in Table S1.

The four calibrated quantities $\theta=(c_m, c_{d,0}, c_{d,1}, C_{spread})$ were obtained by minimizing the combined middle- and downstream-temperature residuals over all 110 FEA cases:

$$\theta^* = \underset{\theta}{\operatorname{argmin}} \sum_{n=1}^{N} \left[(T^n_{m,\mathrm{ROM}} - T^n_{m,\mathrm{FEA}})^2 + (T^n_{d,\mathrm{ROM}} - T^n_{d,\mathrm{FEA}})^2 \right] \tag{S16}$$

After calibration, the ROM-calculated temperature rises relative to T_u were matched to the three thermistor measurements to predict k_s and V_f simultaneously:

$$(k_{s,\mathrm{pred}}, V_{f,\mathrm{pred}}) = \underset{k_s, V_f}{\operatorname{argmin}} \left[(\Delta T_{m,\mathrm{ROM}} - \Delta T_{m,\mathrm{meas}})^2 + (\Delta T_{d,\mathrm{ROM}} - \Delta T_{d,\mathrm{meas}})^2 \right] \tag{S17}$$

where the two temperature rises are referenced to the measured upstream temperature. During prediction, c_d was updated from the current velocity estimate and the bounded minimization was repeated until the velocity changed by less than 10^{-5} m/s or ten outer iterations were completed.

Table S1. Geometric, thermophysical, boundary, and calibrated parameters used in the FEA and reduced-order thermal-resistance model.

Parameter	Symbol	Value and unit	Determination
**Operating and boundary conditions**			
Tissue thermal conductivity	k_s	0.20–0.40 W/(m·K)	Parametric FEA sweep
Mean blood velocity	V_f	0.01–0.10 m/s	Parametric FEA sweep
Heater power	Q	0.05 W	Prescribed heat input
Blood and deep-tissue temperature	T_b	37 °C	Thermal boundary condition
Ambient temperature	T_e	25 °C	Thermal boundary condition
External natural-convection coefficient	h_e	5 W/(m²·K)	Matched to the FEA boundary
**FEA geometry and blood properties**			
Tissue-domain dimensions	$L \times W \times H$	50×20×10 mm³	FEA geometry
Heater radius	r_h	2.0 mm	Biosensor geometry
Thermistor radius	r_s	0.5 mm	Biosensor geometry
Vessel radius	r_v	1.3 mm	FEA geometry
Vessel-center depth	h_v	1.7 mm	FEA geometry
Tissue gap above vessel	δ	0.4 mm	Center depth minus vessel radius
Vessel diameter	D_v	2.6 mm	Twice the vessel radius
Blood density	ρ_f	1060 kg/m³	Blood property at 37 °C
Blood dynamic viscosity	μ_f	0.0035 Pa·s	Blood property at 37 °C
Blood specific heat	$c_{p,f}$	3770 J/(kg·K)	Blood property at 37 °C
Blood thermal conductivity	k_f	0.50 W/(m·K)	Blood property at 37 °C
**Effective ROM geometry**			
Upstream and downstream lateral lengths	L_u; L_d	6.0; 2.7 mm	Sensor-center spacing
Lateral-resistance fit slopes	$S_{u\text{–}m}$; $S_{m\text{–}d}$	2006.25; 536.82 m⁻¹	FEA heat-flux fitting
Effective lateral areas	$A_{u\text{–}m}^{lat}$; $A_{m\text{–}d}^{lat}$	2.99; 5.03 mm²	Figure S1
Total active areas	A_m; A_d	30; 15 mm²	FEA region above 37 °C
Vessel-coupled areas	$A_{w,m}$; $A_{w,d}$	14.25; 10.06 mm²	FEA cross-sectional partition
Effective bypass areas	$A_{m,b}^{eff}$; $A_{d,b}^{eff}$	15.75; 4.94 mm²	Figure S2
Bypass conduction lengths	$L_{m,b}$; $L_{d,b}$	3.0; 3.0 mm	Maximum active thermal depth
Middle-node axial location	x_m	4.14 mm	Hausen-correlation interval
Downstream segment limits	$x_{d,1}$; $x_{d,2}$	4.14; 5.24 mm	Hausen-correlation interval
**FEA-calibrated ROM parameters**			
Middle-node convection factor	c_m	0.5907	Global fit to 110 FEA cases
Downstream convection intercept	$c_{d,0}$	0.0318	Global fit to 110 FEA cases
Downstream velocity coefficient	$c_{d,1}$	0.1246	Global fit to 110 FEA cases
Spreading-resistance coefficient	C_{spread}	9.9283 m⁻¹	Global fit to 110 FEA cases

Note: Paired values are listed in the same order as their paired symbols. Blood properties were evaluated at 37 °C. The velocity correction in Eq. (S10) uses 1 m/s as the reference velocity.

Table S2. Five-fold cross-validation performance of the candidate machine-learning models.

Model	k_s MAE	k_s RMSE	k_s R^2	V_f MAE	V_f RMSE	V_f R^2	Mean NRMSE (%)
P3 + Ridge	0.000405	0.000487	0.99994	0.000193	0.000264	0.99992	0.269
P2 + Ridge	0.002173	0.002460	0.99849	0.001078	0.001317	0.99790	1.347
SVR (RBF)	0.001101	0.005677	0.99194	0.000567	0.002234	0.99395	2.660
Extra Trees	0.004055	0.006461	0.98956	0.003217	0.004499	0.97546	4.115
kNN (distance weighted)	0.007727	0.011104	0.96918	0.003383	0.004669	0.97358	5.370
Random Forest	0.008427	0.011275	0.96822	0.005055	0.006491	0.94893	6.425
Linear regression	0.011001	0.012575	0.96047	0.005488	0.006723	0.94522	6.879

Note: MAE and RMSE are reported in W/(m·K) for k_s and in m/s for V_f. NRMSE was normalized by the full sampled range of the corresponding output. P2 and P3 denote second- and third-order polynomial ridge regression, respectively. Model settings were Ridge $\alpha = 10^{-6}$; kNN, three distance-weighted neighbors; SVR, $C = 10$, $\varepsilon = 10^{-4}$, and γ = scale; Extra Trees and Random Forest, 300 trees. A random seed of 42 was used. Models are ordered by mean NRMSE.

Table S3. Independent off-grid validation performance of the selected machine-learning inverse model.

Output	MAE	RMSE	NRMSE (%)	R^2
	**Mean [95% interval]**	**Mean [95% interval]**	**Mean [95% interval]**	**Mean [95% interval]**
k_s	0.000455<br>[0.000267, 0.000764]	0.000886<br>[0.000322, 0.001453]	0.443<br>[0.161, 0.726]	0.99976<br>[0.99932, 0.99997]
V_f	0.000762<br>[0.000219, 0.001512]	0.001985<br>[0.000398, 0.003170]	2.205<br>[0.442, 3.522]	0.99424<br>[0.98565, 0.99966]

Note: The model was trained only on the original 110 FEA cases and evaluated using 30 independent off-grid FEA cases, with no model refitting or reselection. The reported intervals are the 2.5th–97.5th percentiles from 10,000 case-level bootstrap resamples. NRMSE was normalized by the full sampled range of the corresponding output. MAE and RMSE are reported in W/(m·K) for k_s and in m/s for V_f.

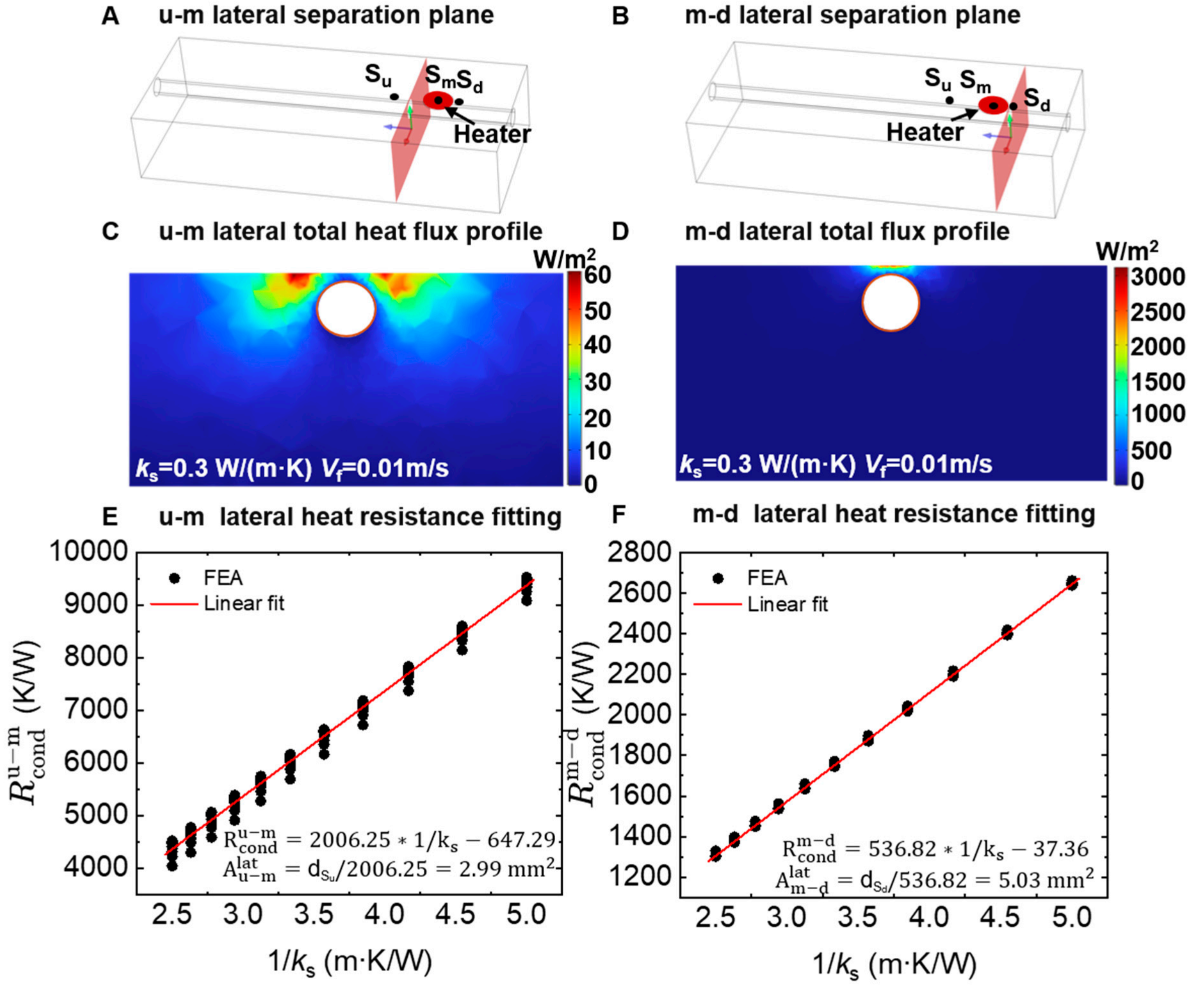

Figure S1. FEA-based calibration of lateral conductive thermal resistance. A) Lateral separation plane for the u-m node pair. B) Lateral separation plane for the m-d node pair. C) Representative lateral total heat flux distributions across the u-m separation planes at $k_s = 0.3$ W/(m·K) and $V_f = 0.01$ m/s. D) Representative lateral total heat flux distributions across the m-d separation planes at $k_s = 0.3$ W/(m·K) and $V_f = 0.01$ m/s. E) Linear fitting of the FEA-derived conductive thermal resistance versus $1/k_s$ for the u-m directions. The fitted slopes are 2006.25 m^{-1} and the effective lateral area is 2.99 mm^2. F) Linear fitting of the FEA-derived conductive thermal resistance versus $1/k_s$ for the m-d directions. The fitted slopes are 536.82 m^{-1} and the effective lateral area is 5.03 mm^2.

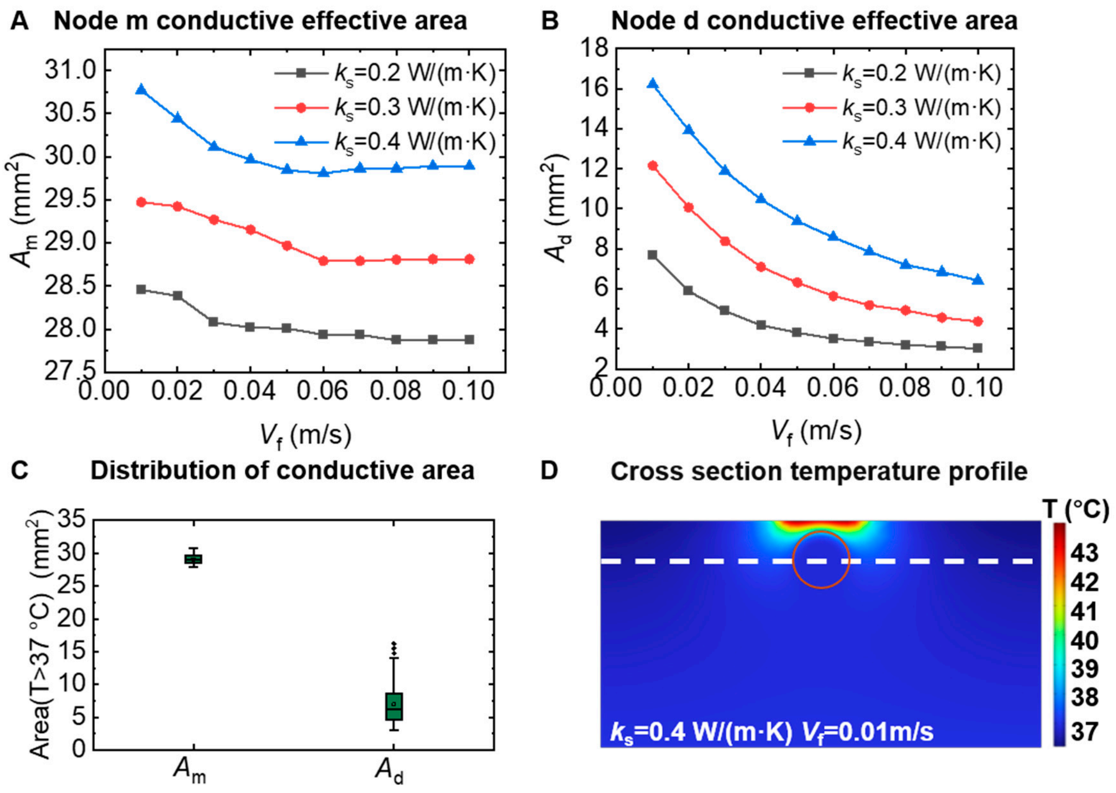

Figure S2. Effective conductive areas for bypass resistance estimation. A) Node m effective area (T>37°C) A_m distribution. B) Node d effective area (T>37°C) A_d distribution. C) distribution of effective conductive area for node m and node d. D) Estimation of the bypass conduction depth from the cross-sectional temperature field, where the thermally active region is defined by T>37°C. Based on the FEA surface integration results, the representative conductive areas were estimated as A_m=30 mm^2, A_d=15 mm^2. The Bypass conduction length was approximated as $L_{m,b}$=$L_{d,b}$=h_v+r_v=3 mm.

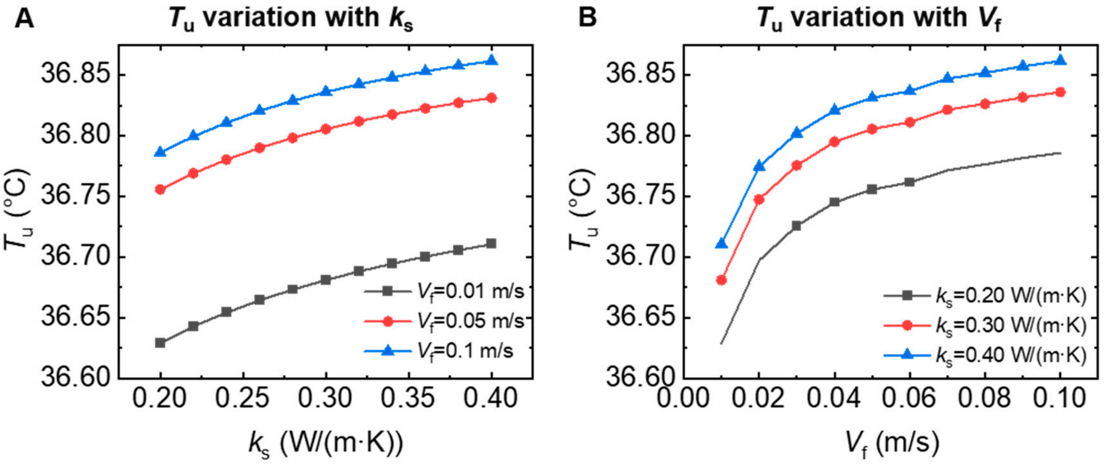

Figure S3. T_u Temperature distribution. A) T_u variation with k_s by FEA. B) T_u variation with V_f by FEA. The temperature change is less than 0.3 °C, so Node u is modeled as a lumped isothermal node.
